# Mutation of *SIVA*, a candidate metastasis gene identified from clonally related bilateral breast cancers, promotes breast cancer cell spread *in vitro* and *in vivo*

**DOI:** 10.1371/journal.pone.0302856

**Published:** 2024-05-09

**Authors:** Anke Vermehren-Schmaedick, Myron Peto, Wendy Wagoner, Kami E. Chiotti, Elizabeth Ramsey, Xiaoyan Wang, Shauna Rakshe, Jessica Minnier, Rosalie Sears, Paul Spellman, Shiuh-Wen Luoh

**Affiliations:** 1 Veterans Administration Portland Health Care System, Portland, Oregon, United States of America; 2 Knight Cancer Institute, Oregon Health & Science University, Portland, Oregon, United States of America; 3 Division of Hematology and Medical Oncology, Oregon Health & Science University, Portland, Oregon, United States of America; 4 Molecular and Medical Genetics, Oregon Health & Science University, Portland, Oregon, United States of America; 5 Knight Cancer Institute, Biostatistics Shared Resource, Oregon Health & Science University, Portland, Oregon, United States of America; 6 Brenden-Colson Center for Pancreatic Care, School of Medicine, Oregon Health & Science University, Portland, Oregon, United States of America; 7 University of California Los Angeles, Los Angeles, California, United States of America; Dr. Anjali Chatterji Regional Research Institute for Homeopathy, INDIA

## Abstract

Metastasis is the most dreaded outcome after a breast cancer diagnosis, and little is known regarding what triggers or promotes breast cancer to spread distally, or how to prevent or eradicate metastasis effectively. Bilateral breast cancers are an uncommon form of breast cancers. In our study, a percentage of bilateral breast cancers were clonally related based on copy number variation profiling. Whole exome sequencing and comparative sequence analysis revealed that a limited number of somatic mutations were acquired in this “breast-to-breast” metastasis that might promote breast cancer distant spread. One somatic mutation acquired was *SIVA-D160N* that displayed pro-metastatic phenotypes *in vivo* and *in vitro*. Over-expression of *SIVA-D160N* promoted migration and invasion of human MB-MDA-231 breast cancer cells *in vitro*, consistent with a dominant negative interfering function. When introduced via tail vein injection, 231 cells over-expressing *SIVA-D160N* displayed enhanced distant spread on IVIS imaging. Over-expression of *SIVA-D160N* promoted invasion and anchorage independent growth of mouse 4T1 breast cancer cells *in vitro*. When introduced orthotopically via mammary fat pad injection in syngeneic Balb/c mice, over-expression of *SIVA-D160N* in 4T1 cells increased orthotopically implanted mammary gland tumor growth as well as liver metastasis. Clonally related bilateral breast cancers represented a novel system to investigate metastasis and revealed a role of *SIVA-D160N* in breast cancer metastasis. Further characterization and understanding of SIVA function, and that of its interacting proteins, may elucidate mechanisms of breast cancer metastasis, providing clinically useful biomarkers and therapeutic targets.

## Introduction

Development of metastatic disease is the most dreaded outcome for breast cancer. When breast cancer becomes metastatic, it is virtually incurable, and treatment is primarily palliative. Despite advances in early detection and treatment in the adjuvant settings, breast cancer patients sometimes develop metastatic disease. This is an important area with unmet need. Metastasis is a multi-step process encompassing the (1) local infiltration of tumor cells into the adjacent tissue, (2) trans-endothelial migration of cancer cells into vessels known as intravasation, (3) survival in the circulatory system, and (4) subsequent extravasation and proliferation in distant organs leading to colonization [1,2]. Thorough understanding of the molecular underpinnings of breast cancer metastasis is essential for risk stratification and development of therapy.

Bilateral breast cancers, an uncommon form and accounting for 3–5% of all breast cancers cases, happens when women develop breast cancers in both breasts, with the second primary cancer in the contralateral breast being either synchronous or metachronous [3–7]. Synchronous bilateral breast cancers are diagnosed 6–12 months apart and represent about 1% of breast cancers [8–10]. Clinically, patients with synchronous bilateral breast cancers behaved similarly to those with unilateral breast cancers [11,12]. However, in metachronous bilateral breast cancers, accounting for 2–3% of breast cancers [7,13], an adjuvant chemotherapy study reported a dual effect on reducing the risk of developing while worsening the prognosis of a second cancer that developed more than one year later [14].

Whole exome sequencing (WES) of primary and contralateral breast cancers showed a high mutational diversity between cancers [15]. The current WES study of 16 sets of bilateral breast cancers revealed that some of them were clonally related, i.e., “breast-to-breast metastasis events”, suggesting that these metastasis events represent initial efforts of primary breast tumors to acquire metastatic/spreading potentials. Since spreading breast cancer cells are returning to the same breast environment these cells have originated from, we speculate that this represents a circumscribed spectrum of cancer metastasis without having to incur extensive genomic changes required for full-blown metastasis. In these “mother to daughter” primary breast tumor pairs, only a limited number of somatic mutations were acquired in the daughter tumors which in essence were metastatic deposits but not primary breast cancers (0–8), suggesting a finite number of mutations were sufficient to orchestrate/initiate metastasis. One such mutation was *SIVA-D160N*.

SIVA apoptosis inducing factor (SIVA) was first identified via a yeast two-hybrid screening with CD27, a member of the tumor necrosis factor receptor family, when it was used as the bait [16]. In previous studies, SIVA, also known as CD27-binding protein (CD27BP), has been demonstrated to play opposite roles: (1) it is activated by the p53 and has pro-apoptotic activities in various cell lines [17,18], (2) it harbors ARF E3 ubiquitin protein ligase activity which leads to p53 degradation through the upregulation of Hdm2 protein [19], (3) *SIVA* ablation prevented the development of non-small cell lung cancer in a mouse model [20] revealing its oncogenic role in the respiratory epithelium, and (4) it can also inhibit STATHMIN, a microtubule destabilizer that when phosphorylated by CamKII may impede EMT and migration [21]. The above are just a few examples that indicate the wide-ranging functions SIVA can play pending on cellular contexts [22].

The current study reports that the *SIVA-D160N* mutation serves as a dominant negative mutation and can promote migration and invasion in breast (and ovarian) cancer cell lines *in vitro* and promote breast tumor growth *in vivo* either as tumor xenografts in immunodeficient mice or an immuno-competent syngeneic mouse model.

## Materials and methods

### Human tissue collection

Tumor specimens included in this study (16 breast cancer pairs, 13 synchronous and 3 metachronous) were derived from the Oregon Health & Science University (OHSU) Knight Cancer Institute Breast Cancer Repository that contained about 2000 samples [23]. These tumors, de-identified and non-traceable, were originally submitted by community hospitals in Oregon, Washington, and Alaska to the College of American Pathologists–certified OHSU Hormone Receptor Laboratory between 1985–1998, in accordance with relevant guidelines and regulations. Breast tumors were snap-frozen within 30 min of biopsy or mastectomy, and continuously maintained frozen at -80°C. All experimental protocols were carried out in accordance with relevant regulations: waiver of informed consent for the study and HIPAA authorization for research was approved by the by the Institutional Review Boards at OHSU and VA and community hospitals in accordance with federal and local privacy laws (OHSU IRB# 00000211, VA IRB# LUOH3850), all medical samples were de-identified and not traceable, last time data were accessed for research purposes was November 2023. Metachronous bilateral breast cancers were defined as bilateral breast cancers that are diagnosed at least 12 months apart. Systematic therapy was given to some patients with metachronous bilateral breast cancers after first cancer was diagnosed. The pathological diagnosis of specimens was confirmed by pathologists.

### Genomic sequencing and analysis

Genomic DNA isolation from the tumors, library construction and sequencing were done by the OHSU Massively Parallel Sequencing Shared Resource on campus (Illumina sequencing) [24] per standard protocols in accordance with institutional regulations and IRB approved protocols. Because of the lack of germline controls, a comparative subtractive analysis was performed between the two tumors from the same patient (right compared with left, and left compared with right) and a subtraction of common variants from the single nucleotide polymorphisms database (dbSNP) and difficult to sequence regions.

In order to detect mutants and discover copy number variants, took HiSeq paired-end reads were aligned to the hg19 human reference genome using bwa mem [25], converting the sam format to bam (binary) format using samtools import (SAMTOOLS, RRID:SCR_002105). After sorting and indexing the reads in the bam formatted file, Picard Tools (MarkDuplicates, [26]) was used to remove duplicate reads generated during the PCR amplification stage. Duplicate removal was done by finding all reads that have identical 5’ coordinates and keeping only the read pair with the highest map quality. After duplicate removal, reads around SNVs and indels were realigned using the GATK Software Library (GATK, RRID:SCR_001876) [27,28]. Local positions to target for realignment were called using RealignerTargetCreator and the reads were realigned using IndelRealigner. Finally, quality scores were recalibrated. This was done using GATK BaseRecalibrator and PrintReads, [29], which binned reads based on the original quality score, the dinucleotide, and the position in the read. To call mutations we compared the tumor samples with the normal samples using muTect [30]. Copy number analyses were done using CNVkit [31]. Briefly, the average coverage per exon as well as off-target regions was found, normalized for the number of reads in the sample and for GC content, and then compared to the normal coverage.

A uniquely acquired mutation in one tumor was defined as having at least 30 independent reads with more than 20% of the reads carrying this mutation in comparison with having at least 10 independent reads over the same genomic segment with fewer than 2% of the reads carrying the same mutation in the contralateral tumor of the same patient. To assess clonal relationship, the copy number segments of different samples was compared using Clonality [32], an R-based package. Likelihood ratios (LR) were generated based on similar gains and losses on chromosome arms. A high LR corresponds to segmentation data from two synchronous tumors that shows greater similarity than those of unrelated tumors and would be evidence of clonality.

### Cell cultures

Human breast (MB-MDA-231 and HCC1954) and ovarian (SKOV3 and OVCAR8) cancer cell lines, as well as mouse 4T1 mammary gland cell lines, were grown in RPMI-1640 (HyClone, Cat# SH30027.01), supplemented with 10% Fetal Bovine Serum (Thermo Fisher Scientific, Cat#26140–079). All cells were grown to approximately 80% confluence prior to splitting/harvesting in a 37°C, 5% CO_2_ tissue culture incubator. Subcultures were made twice a week using 0.05% Trypsin-EDTA. Cell lines were initially obtained from ATCC, except for MB-MDA-231+luciferase (CellBioLabs, Inc Cat# AKR231) and 4T1 (obtained from Dr Sears, OHSU) cells.

### *SIVA* constructs

The human SIVA (NCBI NM_006427.4) open reading frame clone was obtained from GenScript (Cat# OHu18390; pcDNA3.1(+) CMV SIVA P2A-eGFP-neomycin). The wild type SIVA ORF was PCR-amplified (F primer: AAGCTGGCTAGCGTTTAAACTTAAGCTTGC, R primer: CTTGAGCTCGAGAGATCAGGTCTCGAACATGGCACAGCTG; 528 bp), and subcloned into the CMV-IRES-GFP-puromycin vector (Addgene #45567) using the restriction enzymes *Nhe*I and *Sac*I. The SIVA-D160N mutation was introduced by PCR amplification (124 bp fragment) using a gBlock (synthetized by Thermo Scientific) and the Phusion polymerase (F primer: GTGTGTGCGCACCTGCTGGGGCTG, R primer: TTCCTCTAGAAGATCAGGTCTCGAACATGGCACAG; gBlock: CAGTGTGAGCGAGCCCTGTGCGGGCAGTGTGTGCGCACCTGCTGGGGCTGCGGCTCCGTGGCCTGTACCCTGTGTGGCCTCGTGGACTGCAGTAACATGTACGAGAAAGTGCTGTGCACCAGCTGTGCCATGTTCGAGACCTGATCTTCTAGAGGAAGCGGAGCT) and subcloned into the CMV-SIVA-WT-IRES-GFP-puromycin vector using the restriction enzymes *FspA*I and *Xba*I. Both constructs were transformed into NEB-10beta cells (NE BioLabs), grown on LB plates + Ampicillin plates, and sequence-verified.

### Transient and stable transfections (siRNA, shRNA, SIVA constructs)

ON-TARGET plus Human *SIVA* siRNAs were obtained from Dharmacon (#LQ-012262-00-0002; sense sequences #09: GCAGUGACAUGUACGAGAA, #12: CACCAGCUGUGCCAUGUUC), resuspended following manufacturer’s instructions and stored as 20 μM stocks at -20°C. For transient siRNA transfections, 25 nM siRNA + DharmaFECT4 reagent/well (6-well plate) were used, and added to each well when 231L cells were at 50% confluency and incubated in 2 mL serum free media without antibiotics for 48–72 hours in a 37°C, 5% CO_2_ tissue culture incubator. For 231L cells with stable SIVA knockdown, cells were transduced with a lentivirus expressing SIVA shRNA (Dharmacon Smart vector Lenti Cat# V3SH11240-225022166; hCMV promoter, *SIVA* 3’UTR: TAAAAGGCACCCCTCCCGT), selected by cell flow cytometry [33] and maintained with occasional 0.8 mg/mL Hygromycin selection. To generate cell lines overexpressing SIVA-WT and SIVA-D160N proteins, 1 μg of *Ssp*I-linearized vector was used to transfect 231L, HCC1954, SKOV3, OVCAR8, and 4T1 cells using Lipofectamine 3000 Transfection Reagent according to manufacturer’s instructions (Invitrogen, Cat# L3000008). Culture media was changed after 24 hours, and cells were selected with hygromycin (0.8 mg/mL for human cell lines, and 0.2 mg/mL for mouse 4T1 cells).

### Western blot

*Antibodies*: anti-SIVA (Atlas Antibodies Cat# HPA065398, RRID:AB_2685484, pRabbit), anti α-tubulin (Proteintech Cat# 66031-1-Ig, RRID:AB_11042766, mMouse).

Cultured cells were lysed in Cell Lysis Buffer (20 mM Tris-HCl (pH 7.5), 150 mM, NaCl 1 mM, Na_2_EDTA, 1 mM EGTA, 1% Triton X-100) supplemented with HALT protease and phosphatase inhibitor cocktail (Thermo Fisher Scientific, Cat# 78442), incubated on ice for 15 minutes, centrifuged for 10 minutes at 10,000 rpm at 4°C, and the supernatant transferred to a new tube. Total protein concentration was quantified using the Pierce BCA protein assay kit (Thermo Fisher Scientific, Cat# 23225). 40 μg of protein were denatured in reducing buffer, heated to 95°C for 10 minutes, and run on a AnyKD gel (Bio-Rad, Cat# 456–9035). Following SDS/PAGE gel electrophoresis, proteins were transferred to nitrocellulose membranes, which were probed for SIVA (1:1000), and α-tubulin (1:2000). Fluorescent protein-conjugated secondary antibodies (LI-COR Biosciences IRDye®800CW-donkey-αrabbit, Cat# 926–32213, RRID:AB_621848; IRDye®680RD-donkey-αmouse, Cat# 926–68072, RRID:AB_10953628) were then used for signal detection. Visualization was carried-out with the LI-COR Odyssey Infrared Imaging System Scan, with a resolution of 169 μm (LI-COR). Original uncropped and unadjusted blot images can be found in (S1 File).

### Cell proliferation, migration and invasion assays

Cancer cell proliferation *in vitro* was determined by the Trevigen TACS®XTT Proliferation Assay (R&D Systems, Cat# 4891-025-K) according to manufacturer’s instructions. Four 96-well plates were prepared/experiment, with 1500 (231L) or 750 (4T1) cells seeded in each well (100 μL volume) in triplicates, and proliferation measured at day 0, 2, 3 and 4. Cell migration and invasion (each in triplicates) were examined using the Boyden Chamber system (8 μm pore size, Fisher Scientific, Cat# 353097) and the cell Matrigel invasion system (8 μm pore size, Corning, Cat #354480) respectively. Briefly, 300K cells/well (6-well plate) were grown overnight in media + 10% FBS in a 37°C + 5% CO_2_ incubator, then washed with PBS, trypsinized, resuspended in media + 0.1% FBS, filtered (40 μM), and counted. For the migration assay, 43500 cells in 300ul media + 0.1% FBS were carefully added to the migration inserts, placed in warmed 24-well plates containing 750 μl media + 10% FBS/well, and incubated for 6 hours at 37°C + 5% CO_2_. For the invasion assay, 37500 cells in 300 μl media + 0.1% FBS were added to pre-warmed inserts and incubated in 750 μl media + 10% FBS for 24 hours at 37°C + 5% CO_2_. Following incubations, migration and invasion inserts were transferred into Crystal violet solution for 20 minutes at RT to fix and stain cells, and then rinsed thoroughly in beakers with di-ionized water. Inserts were carefully cleaned on the inside (Q-tips) to remove excess of cells that did not migrate, without touching the outside of the mesh (where the cells migrated to). Inserts were let dry overnight, imaged (4 images/insert), and cells counted (for each experiment, cells for 4 images were added up).

### Animals and housing

This study was carried out in strict accordance with the recommendations in the Guide for the Care and Use of Laboratory Animals of the National Institutes of Health. The use and care of animals used in this study follows the guidelines of the VA Institutional Animal Care and Use Committee (Ethics approval code 3181–17, to SWL), and the study is in accordance with the ARRIVE guidelines (https://arriveguidelines.org). All efforts were made to minimize suffering: 1) procedures were performed under isoflurane anesthesia, 2) if a tumor measured larger than 1.5 cm^3^, or significant changes in body weight, body posture or reduced mobility were observed, this animal would be euthanized, 3) euthanasia was done by CO_2_ exposure according to IACUC guidelines.

Single mice were maintained in standard cages at 21°C, on a daily 12-hour light/dark cycle, with food and water *ad libitum*. Fifteen 6-week-old female immune-deficient NOD-scid (NSG; stock #005557) and thirty 6-week-old Babb/c (stock # 028) mice were obtained from JAX and Charles River Laboratories respectively. All animals were acclimated for one week, then ranked by weight, and distributed into three groups (group one: 1,4,7,10,13; group two: 2,5,8,11,14; group three: 3,6,9,12,15). NOD-scid pilot study was done once (n = 5 / group; total n = 15), and Balb/c study was done twice to increase power (n = 5 / group, total n = 30) following a statistician consultation.

### Tail vein injections (MB-MDA-231L), IVIS imaging and analysis

Empty vector (control), SIVA-WT and SIVA-D160N stably transfected MB-MDA-231L (luciferase, 231L) cell cultures were trypsinized, resuspended in ice-cold sterile PBS, filtered through a 40 μm cell strainer and quantified. 7-week-old NOD-scid mice (19.12±0.34 g/animal) were put under a red light for 10 minutes to dilate their tail veins, and then immediately injected into the lateral tail vein with 200 μL PBS containing 25K cells. Injections were performed by an experienced investigator, who randomly assigned the cells to each group. Luciferase-expressing cell spread was followed biweekly (Tuesdays and Thursdays) starting 5 days post-injection, and for a total of 3 weeks. For IVIS imaging, mice were injected with 100 μL of D-luciferin potassium salt (1.5 mg per 10 g animal in 100 μL sterile PBS; Goldbio, Cat# LUCK-1G) and 3 minutes after luciferin injection, the mice were anesthetized using inhaled isoflurane and positioned ventral side up on a heated platform. IVIS imaging was performed by an investigator blinded to the treatment. Bioluminescent images (exposure times were 3s and 60s) were obtained using an IVIS Spectrum CT and processed using Living Image software (RRID:SCR_014247; Perkin Elmer). All animals were euthanized by CO_2_ inhalation after the last IVIS imaging. No animals were lost in the study nor excluded.

### Fat-pad injections (4T1), tumor measurements and analysis

Empty vector (EV as control), *SIVA-WT* and *SIVA-D160N* stably transfected 4T1 cell cultures were trypsinized, resuspended in ice-cold sterile PBS, filtered through a 40 μm cell strainer and quantified. 7-week-old Balb/c mice (17.14±0.29 g/animal) were anesthetized with isofluorane injected with 200 μL of PBS:matrigel (1:1; Matrigel) containing 25K cells into the fourth mammary gland fat pads. Injections were performed by an experienced investigator, who randomly assigned the cells to each group. Tumor formation was monitored thrice weekly (Monday, Wednesday, Friday) starting 5 days post-injection using a caliper (length and width), by two alternating investigators, for a total of 3 weeks. To minimize animal suffering, if a tumor measured larger than 1.5 cm^3^, or significant changes in body weight, body posture or reduced mobility were observed, this animal would be euthanized. At the end of the study, and the tumors were dissected out, measured, fixed in formalin for 24 hours and stored in 70% ethanol at 4°C until paraffin embedding. 5 μm thick sections from each representative specimen were obtained and processed for H&E and immunostaining. All animals were euthanized by CO_2_ inhalation after the last tumor measurement (21 days post-injection). No animals were lost in the study nor excluded.

### Immunocytochemistry (ICC) and Immunohistochemistry (IHC)

*Antibodies*: anti-SIVA (Atlas Antibodies Cat# HPA065398, RRID:AB_2685484, pRabbit), Ki67 (Ventana Medical Systems Cat# 790–4286, RRID:AB_2631262). ICC (SIVA) was done on 231L and 4T1 cell cultures grown on 8 mm coverslips in 24-well plated, fixed for 20 minutes with 4% paraformaldehyde/4% sucrose in PBS at RT and followed by washes in PBS. Cells were permeabilized with 0.3% Triton X-100 in PBS for 5 minutes at RT, followed by PBS wash. Slides were incubated in blocking buffer (0.5% BSA in 0.1 M Tris/0.15 M NaCl) for 30 minutes at RT, followed by the incubation with anti-SIVA antibody (pRabbit, 1:1000) in 0.1% BSA/0.25% Triton X-100 blocking solution) for 24 hours at 4°C in a humid chamber. Slides were then developed using the Vectastain Elite ABD HRP kit (Vector Labs, Cat# PK-6101) and the ImmPACT DAB chromogen (Vector labs, Cat# SK-4105) kit following manufacturer’s instructions. Cells were counterstained with Hematoxylin according to standard protocols and mounted using Epredia™ Cytoseal™ Mountant XYL (Fisher Scientific, Cat# 22-050-262). IHC (Ki67) on tumor tissues was done as described by the manufacturer’s instructions Briefly, deparaffinization was done in xylene followed by rehydration through a descending series of ethanol, antigen retrieval was done in citrate buffer pH 6 for 1 hour, blocking was done in 5% goat serum protein blocker for 4 minutes, Ki67 antibody incubation was done at @37°C for 16 minutes, and DAB staining for 2 minutes. Finally, Ventana hematoxylin I counterstain was done for 4 minutes, and Ventana bluing reagent counter stain 4 minutes. Slides were mounted using Epredia™ Cytoseal™ Mountant XYL. Images were taken on an AXIO imager.A2 microscope (Zeiss, White Plains, NY, USA), attached to an Axiocam ERc 5s camera, using the ZEN2-blue software. Results (5 images per tumor, 10 tumors for each treatment) were expressed as % of positive Ki67 cells relative to total number of cells (total counts of hematoxylin-stained nuclei).

### TUNEL assay

Apoptotic activity was analyzed TUNEL assay, using the ApopTag® Plus peroxidase *in situ* apoptosis detection kit (EMD Millipore, Cat# S7101) according to the manufacturer’s protocol. The positive TUNEL signals were counted under a AXIO imager.A2 microscopy (Zeiss, White Plains, NY, USA). Methyl green (Sigma, Cat# 67060) was used as counterstain. Apoptotic cells were calculated by averaging the 5 images per tumor (10 tumors for each group).

### Statistical analysis

Results were expressed as mean ± standard error of the mean (SEM). All statistical analyses were performed with SAS software version 9.4 (SAS Institute, Cary, NC), and graphs were generated by R software [34] and GraphPad Prism 9 [35]. Statistical differences between multiple groups were done using one-way ANOVA test followed by Tukey’s or Dunnett’s post-hoc tests (as indicated in figure legends). Statistical differences in tumor growth over time between multiple groups was done using two-way ANOVA (mixed effects) test followed by Tukey’s post-hoc test. Statistical differences in cell proliferation were assessed by simple linear regression test (proliferation independent variable is log scale). A *p* value of less than 0.05 was considered statistically significant.

## Results

### Characterization of bilateral breast tumors and identification of the *SIVA D160N* mutation

Sixteen pairs of bilateral breast cancers were identified from a well-annotated breast cancer tissue repository (Knight Cancer Institute) of 2000 total samples (0.8%). Thirteen of them were synchronous and three metachronous. Thirteen pairs existed as frozen samples and three as pulverized. Demographics, histology, clinical staging, biomarker status, laterality, intervals between the detection of the two tumors and assessed clonal relationship are presented in Table 1. The depth of sequencing, i.e., the number of sequencing reads, is also presented in Table 1. Because no germ line controls were available, we performed a comparative subtractive analysis between the two tumors from the same patient (right compared with left and left compared with right) and a subtraction of common variants from the single nucleotide polymorphisms database (dbSNP) and difficult to sequence regions. A uniquely acquired mutation in one tumor was defined as having at least 30 independent reads with more than 20% of the reads carrying this mutation in comparison with having at least 10 independent reads over the same genomic segment with fewer than 2% of the reads carrying the same sequence change in the contralateral tumor of the same patient.

**Table 1:** Bilateral breast cancers used in this study.

ID	breast	seq. depth	age	synch	path	nodes	size	stage	grade	ER	PR	HER2
1013	L	158	84	yes	NOS	ND	ND	ND	ND	+	+	ND
1012	R	164	84		NOS	ND	ND	ND	ND	+	+	ND
83T	L	124	89	yes	NOS	ND	ND	ND	ND	+	+	ND
1563	R	135	89		NOS	ND	ND	ND	ND	+	+	ND
218	L	162	75	yes	NOS	ND	ND	ND	ND	+	+	ND
219	R	140	75		NOS	ND	ND	ND	ND	+	+	ND
608	L	134	91	yes	IDC	0/7	3.0	1	2	+	+	low
609	R	92	91		NOS	ND	ND	ND	ND	+	+	ND
257	L	126	53	yes	IDC/DCIS	0/32	3.0	3	2	+	+	ND
256	R	207	53		IDC/DCIS	10/24	7.0	3	2	+	+	low
7	L	91	48	yes	NOS	ND	ND	ND	ND	+	-	ND
7	R	97	48		NOS	ND	ND	ND	ND	-	-	ND
**8**	**L**	**130**	**35**	**no**	**IDC**	**3/16**	**7.0**	**3**	**2**	**+**	**+**	**ND**
**8**	**R**	**135**	**38**	**32 mo**	**IDC**	**11/14**	**10.0**	**3**	**2**	**+**	**-**	**low**
9	L	128	50	yes	NOS	ND	ND	ND	ND	+	+	ND
159	R	212	50		NOS	ND	ND	ND	ND	+	+	ND
**10**	**L**	**110**	**37**	**no**	**NOS**	**ND**	**ND**	**ND**	**ND**	**+**	**-**	**ND**
**10**	**R**	**143**	**39**	**16 mo**	**IDC**	**12/16**	**7.5**	**ND**	**3**	**+**	**-**	**ND**
14	L	96	81	**no**	NOS	ND	ND	ND	ND	+	+	ND
14	R	88	82	12 mo	NOS	ND	ND	ND	ND	+	+	ND
**15**	**L**	**96**	**62**	**yes**	**NOS**	**ND**	**ND**	**ND**	**ND**	**+**	**+**	**ND**
**15**	**R**	**103**	**62**		**NOS**	**ND**	**ND**	**ND**	**ND**	**+**	**+**	**ND**
16	L	143	47	yes	IDC	0/13	2.5	2	2	+	+	low
16	R	156	48		IDC	0/9	1.4	1	3	-	-	high
17	L	128	52	yes	NOS	ND	ND	ND	ND	-	+	ND
17	R	79	52		NOS	ND	ND	ND	ND	+	+	ND
19	L	168	60	yes	IDC	0/24	1.4	1	3	+	-	ND
19	R	124	60		comedo	0/16	1.3	0	3	ND	ND	low
23	L	228	73	yes	IDC	0/14	1.2	1	1	+	+	low
24	R	193	73		LOB	0/14	1.5	1	ND	+	+	ND
21	L	88	85	yes	LOB	ND	3.5	2	3	+	+	ND
21	R	88	85		mucinous	ND	3.0	ND	3	+	+	low

Breast cancer tumor pairs (L = left, R = Right) were obtained from sixteen patients, each patient pair is clustered in a row separated by lines from other patient tumor pairs. Three tumor pairs were not synchronous (sample ID 8, 10, 14), with the second contralateral tumor detected 32, 16 and 12 months after the first tumor. Rows with bold font indicate patients with tumors that showed strong evidence of clonality. NOS = invasive breast cancer not otherwise specified; IDC = invasive ductal carcinoma; DCIS = ductal carcinoma *in situ*; LOB = invasive lobular cancer; ND = not documented, ER = estrogen receptor; PR = progesterone receptor.

The called variants were examined for overlap with known variants from publicly curated sets of breast cancer samples, and 1098 breast cancer samples from the TCGA-BRCA project located on the GDC Data Portal were used. For the samples in this study, we called variants without matched normal, filtering on dbSNP and requiring 0.20 VAF and 30x coverage, with an extensive exome sequencing coverage of at least 79x (Table 1; S1 Fig in S2 File). Furthermore, variants that were common between three or more samples were filtered out. A comparison of the fraction our samples with commonly mutated genes and the fraction of the TCGA set with those genes mutated is shown in Table A in S2 File. Many of the same genes were found to be mutated in our sample set were mutated in the TCGA-BRCA sample set.

Furthermore, when the *PIK3CA* mutations identified using the opposite tumor as a normal were examined, all mutations were found to be common breast cancer hotspots as identified by COSMIC. These included p.H1047R, p.E542K, and p.M1043I, listed in Table B in S2 File. Both above indicated common molecular profiles were shared between bilateral and unilateral breast cancers.

**Fig 1:**
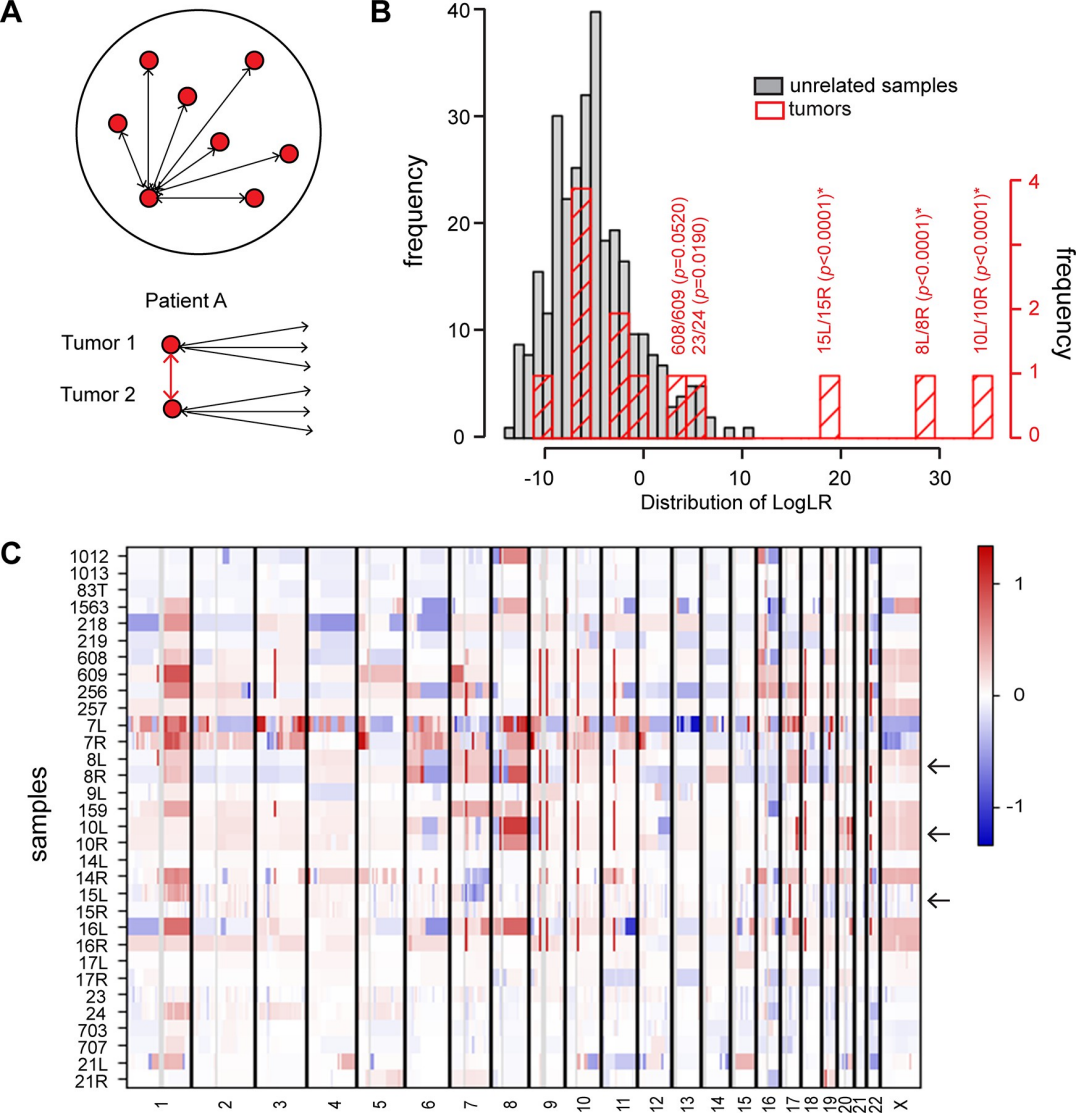
Clonality and copy number profiles. (**A**) Schematic description of the clonality tool. Top: Each red circle represents one breast cancer sample, and following segmentation, the log2 ratios for each sample are compared all against all to generate a set of correlations from known unrelated tumors, with N(N-1)/2 patient comparisons for reference. Bottom: For samples from the same patient, a higher correlation between the two tumors (red arrows) compared to the correlation for unrelated samples (black arrows) is taken as evidence of clonality. (**B**) Clonal relationship of bilateral breast cancers. The distribution of logLR (LR = likelihood ratio) among comparisons of unrelated samples (gray) or tumors from the same patients (red) is shown along the x-axis, while y-axis show the case counts. The positions of three tumor pairs (15L/15R, 8L/8R, 10L/10R) that show strong evidence for clonality are highlighted (*). (**C**) Copy number profiles showing gains (red) and losses (blue) for all the bilateral breast cancers. 15L/15R, 8L/8R, and 10L/10R (arrows) show strong evidence of clonality based on comparison of their copy number segmentation ratio.

Clonal relationship was assessed by comparing the copy number segments of different samples in an all-against-all comparison for all tumors from all patients using the segmentation data (Fig 1A). Likelihood ratios (LR) were generated based on similar gains and losses on chromosome arms. A high LR corresponds to segmentation data from two synchronous tumors that show greater similarity than those of unrelated tumors and would be evidence of clonality, and the three tumor pairs (15L/15R, 8L/8R, 10L/10R) that show strong evidence for clonality are highlighted in Fig 1B. A heat map of copy number variation is shown in Fig 1C, where the 15L/15R, 8L/8R and 10L/10R tumor pairs show strong evidence of clonality based on comparison of their copy number segmentation ratio.

We then compared non-synonymous exonic mutations found in tumors from one side against their contralateral breast tumor from the same patient, identifying unique mutations present in one but not the contralateral tumor in these three patients: 0/0 (pair 10L/10R), 0/5 (pair 8L/8R), 5/8 (pair 15L/15R). In patient 8 the tumor on the right side was detected 32 months after the tumor on the left side, and the unique somatic mutations present on the right tumor were *RYR3* (SNV, c.G7990A, p.E2664K), *DDI1* (SNV, c.C107G, p.A36G), *FCGRT* (stop-gain, c.G155A, p.W52X), *SIVA* (SNV, c.G478A, p.D160N), *SLC28A3* (SNV, c.G202A, p.E68K). The left and right breast tumors found in patient 8 showed strong evidence of clonality and the right sided tumor was found 32 months later than the left side, indicating that right sided tumor was a metastatic deposit from the left sided tumor. For the remainder of this study, we focused solely on the p.D160N mutation of the SIVA apoptosis inducing factor (*SIVA-D160N*), as deep deletion of *SIVA* was reported in 12% (36/301) of metastatic breast cancers [36], and SIVA downregulation promoted breast cancer cell aggressiveness both *in vitro* and *in vivo* [21].

### Downregulation of SIVA in 231L cells significantly enhanced cell migration and invasion

**Fig 2:**
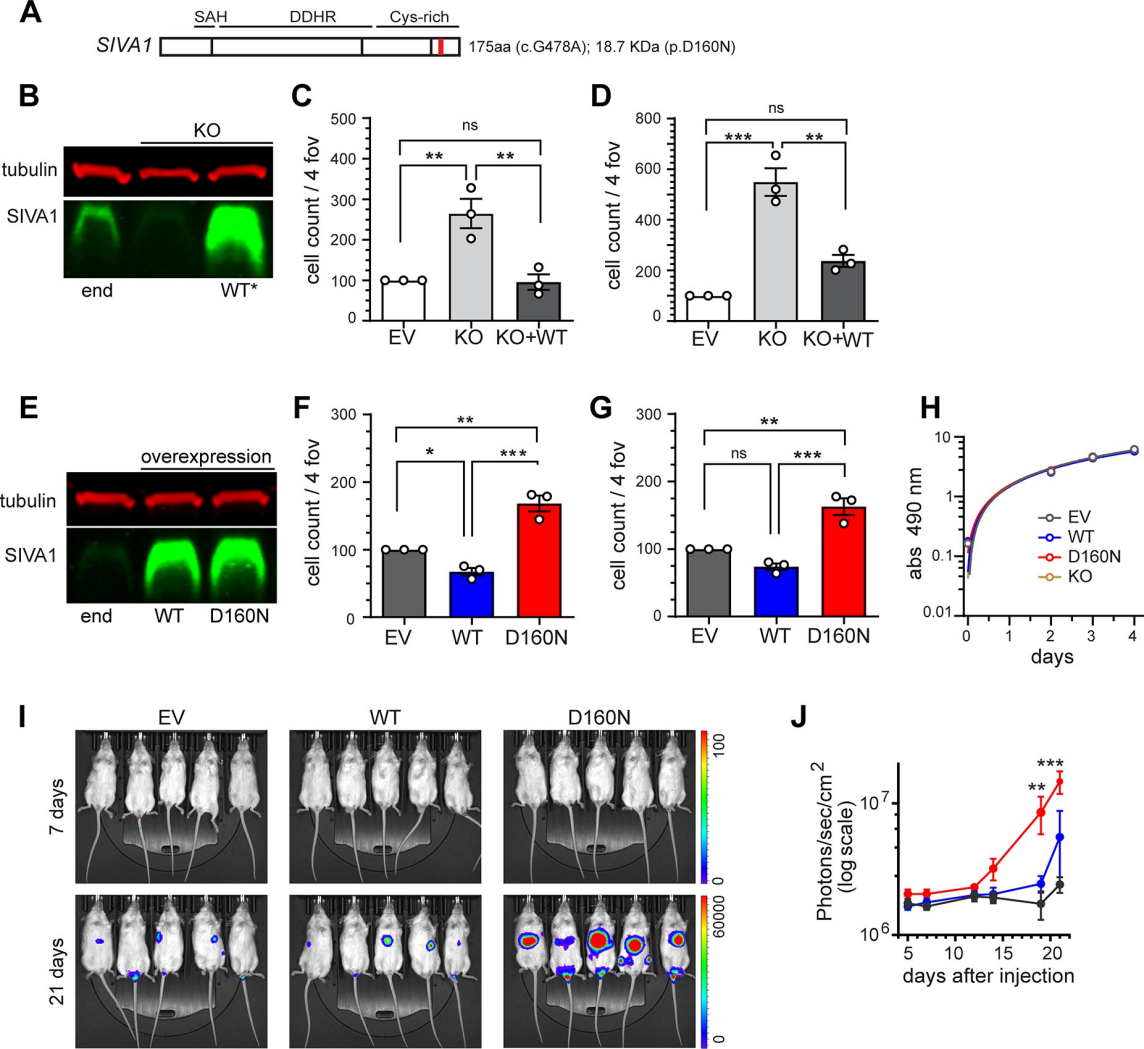
*SIVA* knockdown and *SIVA-D160N* mutation increased migration and invasion capabilities on 231L cells. (**A**) Schematic of SIVA protein showing the position (red line in exon 4) of point mutation identified in the “breast to breast” spread in patient 8. Domains: Amphipathic helix (SAH), death domain homology region (DDHR), cysteine rich domains (Cys-rich). (**B**) Stable knockdown of SIVA protein expression by shRNA (KO) and reintroduction of SIVA-WT protein (WT*), compared to endogenous (end) SIVA expression in empty vector control cells (EV) was demonstrated by Western blot analysis. (**C)** 231L cell migration (Boyden Chamber) and **(D)** 231L cell invasion (Matrigel matrix invasion) analyses were expressed as total cell counts in four fields of view (4 fov) normalized against EV control. Each bar represents the mean ± S.E.M (n = 3, total cells in 4 images/assay); One-way ANOVA (**C**: *p =* 0.0035 and **D**: *p =* 0.0.0002) followed by Tukey’s post-hoc test. (**E**) Overexpression of SIVA-WT (WT) and mutated SIVA (D160N) protein, compared to endogenous SIVA levels in EV control 231L cells was demonstrated by Western blot analysis. (**F**) Cell migration and (**G**) cell invasion analyses of 231L cells overexpressing wild type *SIVA* (WT) and *SIVA* mutation (*D160N*) were expressed as in (**C**, **D**). One-way ANOVA (**F**: *p* = 0.0002, **G**: *p =* 0.0005). (**H**) Proliferation XTT assays, each point represents the mean ± S.E.M (n = 2, 6 replicates/assay); simple linear regression (*p* = 0.8788). (**I**) *SIVA-D160N* expressing 231L cells increased spread of breast cancer cells in immuno-deficient NSG mice. 25K cells were injected into the lateral tail veins of female NSG mice (n = 5), and the proliferation and spread monitored by serial *in vivo* imaging (IVIS). Exposure times are 60s and 3s for 7 days and 21 days respectively. Luminescence counts is indicated in colored bar on the *y*-axis. (**J**) Absolute luminescent intensity of photons emitted from spreading tumor cells in each animal in the IVIS images (**I**) was quantified. Each line represents the mean ± SEM (n = 5). Mixed-effects analysis (*p*<0.0001) followed by Tukey’s post-hoc test. Post Test p values: *p<0.05, **p<0.01, ***p<0.001.

SIVA protein has an amphipathic helical (SAH) region at the N-terminus, a death domain homology region (DDHR) in the middle, and the box-B-like ring finger and two zinc finger-like cysteine rich domain at the C-terminus [37]. Previous studies have identified the SAH and DDHR regions as essential for the pro-apoptotic function of SIVA [38]. The mutation (c.G483A) in *SIVA* led to the substitution of Aspartic acid with the more polar amino acid Asparagine (p.D160N) in exon 4, near the C-terminal region of the protein (Fig 2A). Based on a predictive alphafold model (www.sbg.bio.ic.ac.uk/) due to the lack of a SIVA crystal structure, D160 is located in the Zing-finger domain, forming a D160-K164 salt bridge. The D160N mutation may disrupt this salt bridge, affecting the SIVA protein stability or specificity of protein-protein interactions (S2 Fig in S2 File).

Since cell migration and invasion are hallmarks of cancer cell invasiveness, ([21,39–41], we first verified the role of *SIVA* in breast cancer MB-MDA-231 (expressing luciferase, 231L) cell migration and invasion. For this, we transiently downregulated the endogenous SIVA protein by siRNA transfection, leading to a significant enhancement in the migration and invasion capabilities of the 231L cells (S3 Fig in S2 File).

In addition to generating the transient *SIVA* knockdowns, we used a lentivirus to express shRNA targeting the 3’-UTR of the *SIVA* to make a stable *SIVA*-knockdown (SIVA-KO) 231L cell line (Fig 2B). Migration and invasion of SIVA-KO cells showed a significant increase compared to 231L cells expressing the empty vector (264.8±36.1 and 549.4±54.6 vs 100.0±0.0, respectively; Fig 2C and 2D). Successful re-introduction of SIVA-WT protein was demonstrated by Western blot analysis (Fig 2B) which restored/abolished the migration and invasion phenotypes (95.5±19.2 and 237.4±23.7, respectively) displayed by the SIVA-KO cells (Fig 2C and 2D). Taken together, stable knockdown of *SIVA* in 231L cells significantly enhanced cell migration and invasion, effects that were reversed after re-introducing the expression of *SIVA-WT*. This highly suggests that *SIVA* is involved in the regulation of 231L cell mobility and invasiveness as previously reported in other cell lines such as breast MCF7 and colorectal HCT116 cells [21,42], and now validated in MB-MDA-231 cells.

### Expression of *SIVA-D160N* mutant in 231L cells significantly enhanced cell migration and invasion, as well as tumor spread *in vivo*

We then set out to investigate the potential effect of the *SIVA-D160N* mutation in breast cancer cell proliferation, migration, and invasion, as well as tumor proliferation/spread in NSG-scid female mice. We stably expressed the empty vector (EV, control), *SIVA-WT* (WT) and *SIVA-D160N* (D160N) mutant constructs in 231L cells. The expression of SIVA protein in these cells was verified by Western blot analysis (Fig 2E). Migration analysis revealed a statistically significant increase in the *SIVA-D160N* expressing cells compared to the *SIVA-WT* expressing cells and the EV control (168.2±11.7 vs 67.4±5.6 and 100.0±0.0 respectively; Fig 2F). Invasion analysis revealed a statistically significant increase in the *SIVA-D160N* expressing cells compared to both, *SIVA-WT* and EV expressing cells (162.8±12.5 vs 73.7±5.0 and 100.0±0.0 respectively; Fig 2G). Interestingly, when we measured cell proliferation by XTT assays, simple linear regression analysis found that 231L cells expressing *SIVA-WT* and *SIVA-D160N* mutant displayed similar proliferation rates compared to EV control and SIVA-KO (simple linear regression analysis, *p* = 0.8788) (Fig 2H). Taken together, the expression of *SIVA-D160N* increased migration and invasion in 231L cells similarly to that of ablation of *SIVA* expression (KO), suggesting that *SIVA-D160N* serves as a dominant negative mutation that interferes with the wild type function of SIVA protein in 231L cells.

To investigate whether *SIVA-D160N* conferred increased metastatic potential, we used a xenograft model of metastasis. For this, we introduced 231L cells stably expressing EV (control), *SIVA-WT* and *SIVA-D160N* cells to immuno-deficient female NSG-scid mice via lateral tail vein injections (n = 5). The survival and expansion of these cells were then monitored twice weekly by serial IVIS imaging (Fig 2I). The luminescent intensity of photons emitted from each tumor was quantified, and tumors overexpressing *SIVA-D160N* mutation grew significantly faster than *SIVA-WT* or EV expressing control 231L cells (Fig 2J). Taken together, the above experiments showed that the *SIVA-D160N* mutation displayed metastasis promoting phenotype in breast cancer cell line 231L both *in vitro* and *in vivo*.

### Expression of *SIVA-D160N* mutant in mouse 4T1 cells significantly increased metastasis *in vivo*

**Fig 3:**
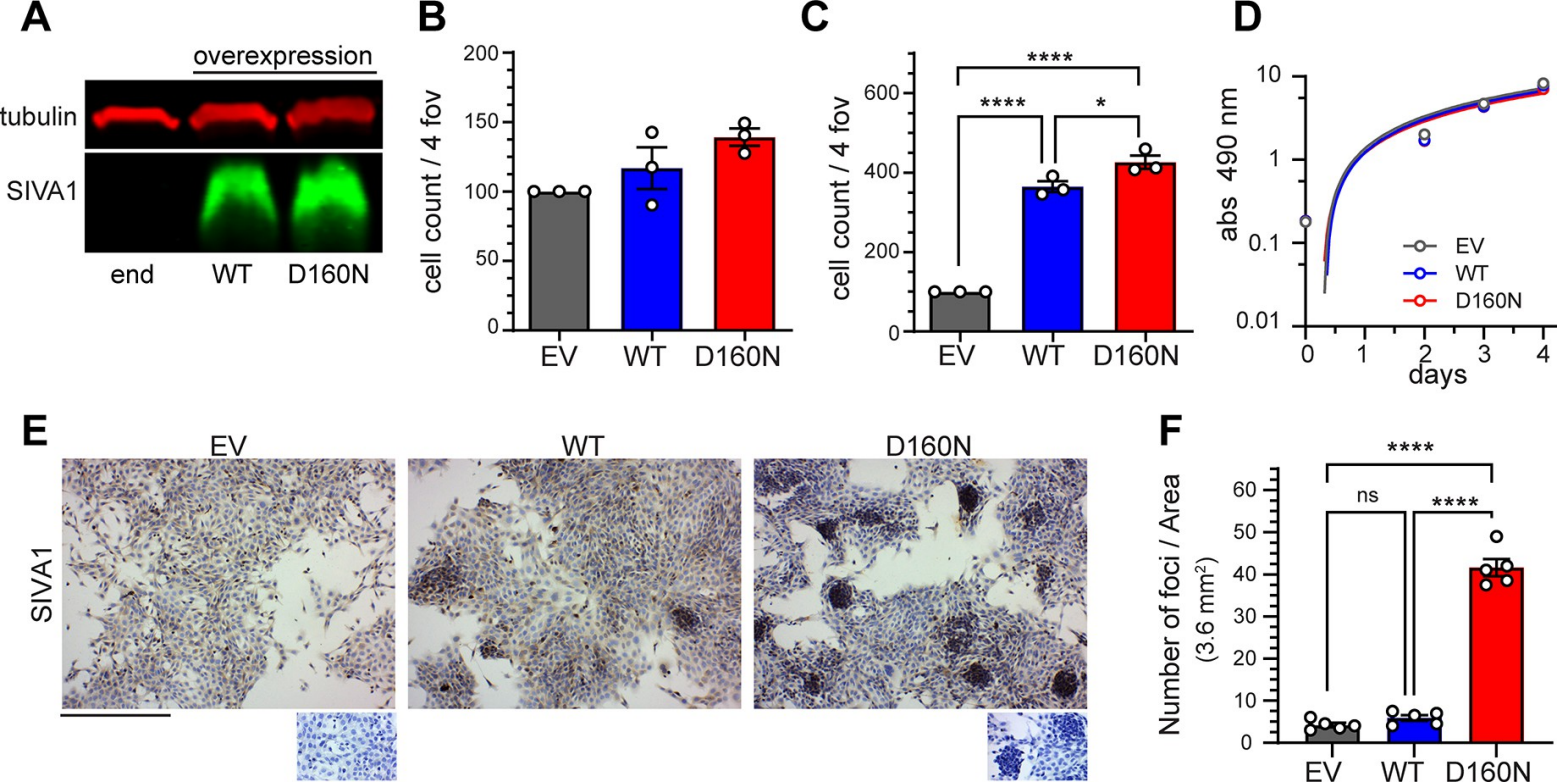
*SIVA-D160N* on cell migration, invasion, and proliferation of 4T1 cells. (**A**) Overexpression of *SIVA* wild type (WT) and *SIVA-D160N* mutant (D160N) in 4T1 cells as compared to empty vector (EV) by Western analysis. (**B**) 4T1 cell migration (Boyden Chamber) and (**C**) 4T1 cell invasion (Matrigel matrix invasion) analyses were expressed as total cell counts in four fields of view (4 fov) normalized against EV control. No differences were observed for cell migration, while *SIVA-WT* and *SIVA-D160N* overexpression were significantly more invasive than EV control. One-way ANOVA (*p* = 0.0682 migration, *p*<0.0001 invasion; n = 3, total cells in 4 images/assay). (**D**) Proliferation of 4T1 cells overexpressing *SIVA-WT* and *SIVA-D160N* was not significantly different from control (EV) 4T1 cells. Each line represents the mean ± SEM (n = 3, 6 replicates/assay); simple linear regression, *p* = 0.096. (**E**) Representative images for SIVA IHC staining of 4T1 cells (top images) and no primary control (small bottom images) showing the increased presence of *SIVA-D160N* expressing foci/aggregates. Scale bar 200 μm. (**F**) Quantification of aggregates in (**E**). **B**, **C**, **F** graphs represent mean ± SEM, One-way ANOVAs were followed by a Tukey’s post-hoc test: *p<0.05, ****p<0.0001.

We then examined the impact of *SIVA-D160N* expression on mouse 4T1 mammary gland cancer cells, using a syngeneic mouse breast cancer model. We overexpressed empty vector (EV as control), *SIVA-WT* or *SIVA-D160N* mutation via plasmid mediated gene transfer in 4T1 cells and obtained polyclonal stable transfectants. The expression of SIVA protein in these 4T1 transfected cells was verified by Western blot analysis (Fig 3A). Migration analysis of 4T1 cells revealed no significant differences among groups (*p* = 0.0682, Fig 3B), while invasion analysis showed that both *SIVA-WT* and *SIVA-D160N* had increased invasion capabilities compared to control, (426.2±16.6 and 364.6±13.6 vs 100.0±0.0 respectively), with *SIVA-D160N* mutant expressing cells were significantly more invasive than WT (Fig 3C). These experiments were done three times with identical findings. Cell proliferation of 4T1 cells expressing *SIVA-WT* or mutant *SIVA-D160N* displayed similar proliferation rates compared to EV control (Fig 3D) by XTT assays and simple linear regression analysis (*p* = 0.960).

Interestingly, while culturing 4T1 cells for five days, we noticed that the cells expressing *SIVA-D160N* formed significantly more small and compacted 3D foci or aggregates (41.60±2.02 aggregates, *vs*. 5.90±0.70 and 4.20±0.51 aggregates for *SIVA-WT* and EV, respectively; n = 10). This is an indication of anchorage-independent cell growth; this behavior was maintained even after filtering the 4T1 cells prior to seeding (Fig 3E and 3F; S4 Fig in S2 File).

**Fig 4:**
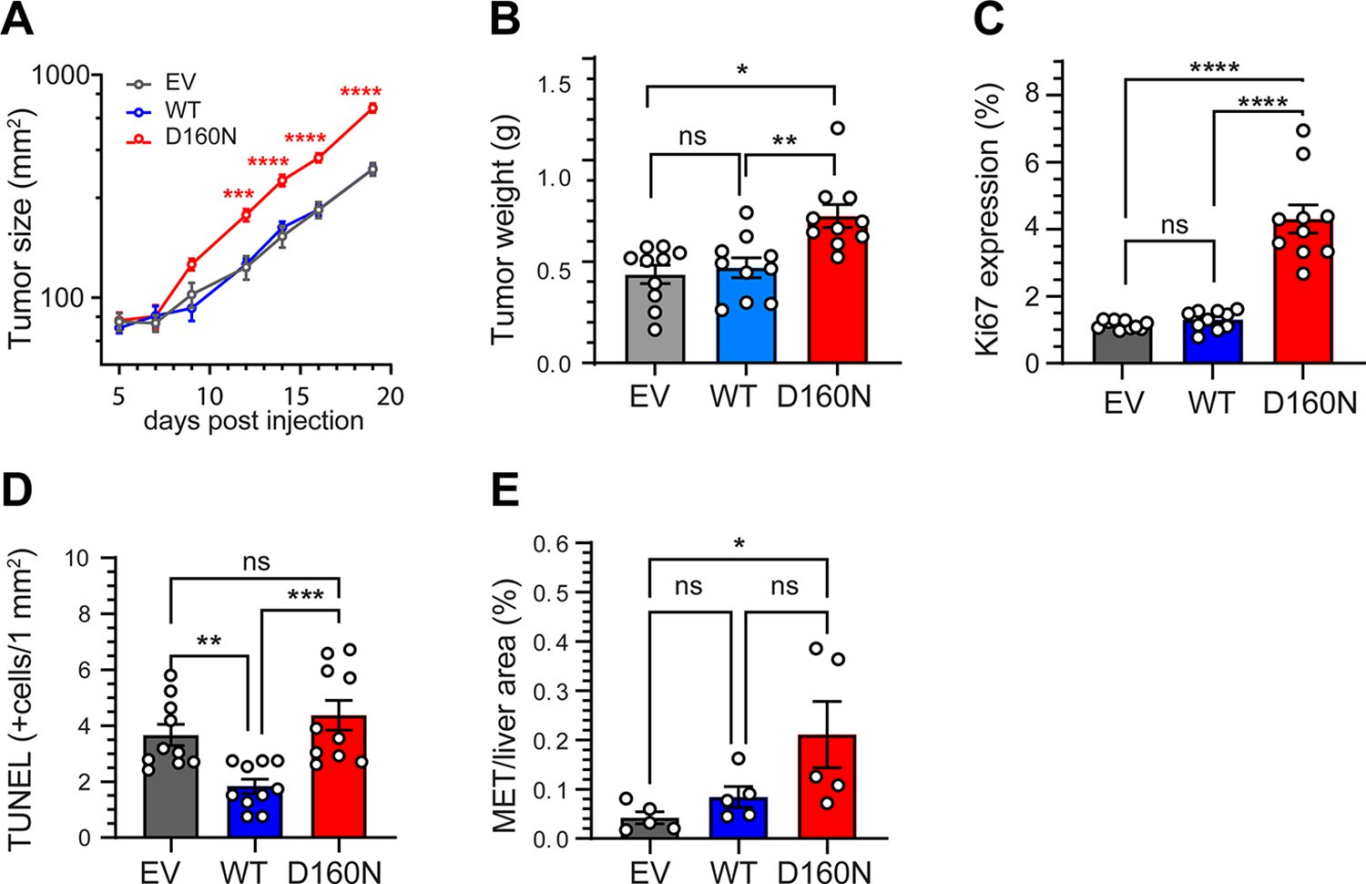
Expression of *SIVA-D160N* mutant in mouse 4T1 increases tumor growth and liver metastasis *in vivo*. **(A)** Orthotopic injection of mammary fat pads with 4T1 cell expressing EV, *WT* or *D160N*, showed tumors expressing *SIVA-D160N* grew the fastest. Results are expressed as the mean ± SEM (n = 10). Mixed-effects analysis (p<0.0001, days post-injection, tumor size and interaction) was followed by a Tukey’s post-hoc test. **(B)** *SIVA-D160N* tumors were significantly larger than EV and *WT* tumors; one-way ANOVA (*p* = 0.0006; n = 10). **(C)** Percentage of Ki67 positive cells per field of view (1 mm^2^). One-way ANOVA (*p*<0.0001; n = 10; 3 images/assay). (**D**) Number of positive cells per area (1 mm^2^) following TUNEL staining. One-way ANOVA (*p* = 0.0004; n = 10; 3 images/assay). **(E)** Total *SIVA-D160N* METs/liver area (%) was significantly higher than in livers with *SIVA-WT* or control EV expressing 4T1 cells (n = 5, 5 images/animal). One-way ANOVA (*p* = 0.0352). **B, C, D, E** graphs represent mean ± SEM, One-way ANOVAs were followed by a Tukey’s post-hoc test: *p<0.05, **p<0.01, ***p<0.001, ****p<0.0001.

We then introduced the 4T1 cell clones via orthotopic mammary fat pad injection into syngeneic female animals of Balb/c background and serially monitored the growth of the mammary gland tumors via a caliper. Mammary fat pad *SIVA-D160N* tumors grew significantly faster compared to EV control and *SIVA-WT* tumors, with the last two growing at identical rates (n = 10, Fig 4A). The size of the *SIVA-D160N* tumors after dissection was also significantly larger (0.724±0.057 g) than 4T1 cells expressing EV or *SIVA-WT* (0.436±0.045 and 0.470±0.049 g, respectively; n = 10) (Fig 4B). There was no difference in the animal weights at the time of dissections (17.64±0.45 (EV), 17.79±0.42 (*WT*) and 17.58±0.44 (*D160N*) g; n = 10, p = 0.9363).

To assess the effect of *SIVA-D160N* mutation in the growth of 4T1 cells, the tumor samples were analyzed by Ki-67 immunostaining. Qualitative microscopic examination Ki-67-stained tumor sections showed an increase Ki-67- positive cells in *SIVA-D160N* expressing tumors as compared with EV control and *SIVA-WT* groups (S5A Fig in S2 File). Quantification of Ki-67 immunohistochemical staining showed significantly more Ki-67-positive cells in *SIVA-D160N* expressing tumors (4.306±0.427%), compared with EV and *SIVA-WT* expressing cells (1.153±0.040% and 1.299±0.090%, respectively; n = 10; Fig 4C).

In addition, we determined apoptosis in these tumors by TUNEL assay to identify cells with nuclear DNA fragmentation. Cells were scored as apoptotic when they showed brown staining (TUNEL positive), compared to the methyl green counterstaining (TUNEL negative) (S5B Fig in S2 File). Quantification of the number of apoptotic cells showed a significant decrease in the TUNEL-positive cells in the tumors expressing *SIVA-WT* (1.836±0.255%) compared with control (EV) and *SIVA-D160N* expressing cells (3.667±0.382% and 4.372±0.531%, respectively; n = 10; Fig 4D).

Mouse 4T1 cancer cells can form visible metastatic nodules in the lung and liver tissues. While we did not detect metastasis in the lungs, livers from control EV, *SIVA-WT* and *SIVA-D160N* expressing 4T1 cell tumor-bearing mice showed presence of metastatic deposits macroscopically (not shown). We examined the liver sections stained with H&E microscopically. We expressed the area occupied by the total metastatic lesions area relative to the liver area examined, with the livers from *SIVA-D160N* expressing tumors being significantly larger (0.211±0.068%) compared to EV and *SIVA-WT* expressing tumors (0.042±0.012% and 0.084±0.021%, respectively; *p* = 0.0352; n = 5; Fig 4E; S5C Fig in S2 File).

### *SIVA-D160N* mutation increases migration and invasion in additional breast (HCC1954) and ovarian (SKOV3, OVCAR8) cancer cells

Though the incidence of single nucleotide mutation in *SIVA* in breast cancer was low, a survey of The Cancer Genome Atlas (TCGA; [43]) via the Genomic Data Commons Data Portal found that 19% (204/1063) of primary breast cancers and 23% (129/560) of primary ovarian cancers had loss of copy numbers of *SIVA*. Other tumor types that had copy number loss of greater than 30% of the cases included cholangiocarcinoma (55.6%, 20/36), mesothelioma (40.7%, 35.86), renal clear cell carcinoma (37.3%, 193/517), and rectal adenocarcinoma (35.3%, 59/167). The above suggested a more global role of *SIVA* in cancer biology.

As SIVA is known to interact with multiple signaling molecules, its copy number reduction may have pleiotropic effects in cancer biology by affecting myriad interactions simultaneously. In this regard, *SIVA-D160N* mutation represents a rare opportunity when a circumscribed spectrum of pathways is expected to be affected by a single amino acid substitution, affording a unique opportunity to study the signaling pathway(s) governed by *D160N* in *SIVA* that mediated increased breast cancer aggressiveness *in vivo* and *in vitro*.

To broadly assess the functional outcome of over-expressing *SIVA-WT* and *SIVA-D160N* compared to control (EV) across multiple cell lines, additional stable polyclonal transfectants of these expression vectors were achieved in human breast cancer (HCC-1954) and ovarian cancer (SKOV3 and OVCAR8) cell lines. Western analysis confirmed the expression of the respective proteins in stable transfectants (S6A Fig in S2 File). Over-expression of *SIVA-WT* significantly decreased migration in all three cell lines when compared to control EV (S6B Fig in S2 File). *SIVA-D160N* expressing cells increased invasion (S6C Fig in S2 File) when compared when compared with EV expressing cells, respectively in all three cell lines as in 231L. In summary, a direct comparison between *EV* and *SIVA-D160N* overexpression consistently showed a significant increased invasion in the cell expressing the *SIVA-D160N* across four human cancer cell lines tested (and three for migration). These observations support a broader involvement of wild type *SIVA* and *D160N* mutation in the aggressiveness of cancer cells. We have performed RNAseq experiments to understand the signaling pathways downstream of *SIVA-D160N* mutation. Due to the exploratory nature of these experiments, the results are presented in the supplemental section (S3 File).

## Discussion

Synchronous bilateral breast cancer cases (as defined by diagnoses less than two months apart) had worse breast cancer specific survival than the unilateral breast cancer cohort because of the higher-risk cancers of the bilateral breast cancer cases. In a matching analysis, breast cancer specific survival was equivalent for the synchronous bilateral breast cancer cases and their high-risk matches. Thus, for a patient with synchronous bilateral breast cancers, an appropriate systemic therapy selection can be made by considering the prognosis of their higher-risk cancer [44]. Multiple somatic mutations that were commonly described in sporadic unilateral breast cancers were also observed in bilateral breast cancers in the present study (Table A in S2 File). The above clinical and genetic analyses indicate bilateral breast cancers share similar biology and clinical behaviors as unilateral breast cancers.

SIVA is a proapoptotic protein originally identified by virtue of its interaction with CD27 and other death receptors [16,45]. The current literature suggests SIVA is pro-apoptotic and capable of inhibiting the growth of malignant tumors in breast [21], cervical [39,46], ovarian [40], colorectal [47] and acute leukemia [48] cancers, but anti-apoptotic and carcinogenic in non-small cell lung cancer [20], osteosarcoma [49] and gastric [38] cancers. SIVA therefore has a role as pro- or anti-malignant factor, depending on the cellular context.

In studies done using cell lines, SIVA was shown to be a direct p53 target gene that is specifically upregulated relative to G1 cell-cycle arrest to promote apoptosis [17,18], suggesting that SIVA may itself have a tumor-suppressor function. On the other hand, SIVA has been reported to suppress p53 activity by stabilizing the interaction between MDM2 and p53, leading to increased p53 ubiquitination and degradation, and increased bromodeoxyuridine (BrdUrd) incorporation [19,49], indicating that SIVA could also play a tumor-promoting role, further validating that the role of SIVA varies with cellular context.

Earlier studies using animal models showed that conditional inactivation of *Siva* in an autochthonous oncogenic KRASG12D–driven mouse model for non-small cell lung cancer (NSCLC) decreased tumor numbers and tumor burden relative to *Siva*-expressing controls. The *SIVA* knockdown also inhibited proliferation and transformation in both mouse and human NSCLC cells *in vitro*. The enhancement of tumorigenesis by SIVA relates to its ability to promote mTOR signaling, inhibit autophagy, and augment metabolic activity [20]. On the contrary, nude mice injected with MCF7-SIVA shRNA cells exhibited markedly increased tumor metastasis compared to mice injected with MCF7-control shRNA cells. Analysis of tumors in lungs and livers further verified the highly enhanced metastatic potential of MCF7-SIVA shRNA cells [21]. This study showed that SIVA counteracted STATHMIN, an important regulator for microtubule dynamics, and defined a role for SIVA in the suppression of EMT and tumor metastasis [21]. A subsequent study showed that the overexpression of SIVA inhibited proliferation, promoted apoptosis and suppressed migration and invasion by facilitating phosphorylation of Stathmin and polymerization of α-tubulin in ovarian and cervical cancer cells [39,40].

Our current study suggested that *SIVA-D160N* functioned as a dominant negative mutation. This is based on the observation that over expression of wild type *SIVA* in human breast and ovarian cancer cell lines generally suppressed the migration and/or invasion; and over-expression of *SIVA-D160N* enhanced migration and/or invasion, in a manner like what we expect with *SIVA* knockdown with shRNA over expression. The signaling function(s) that are dependent on the aspartic acid at the position 160 of the wild type SIVA protein appeared to impart an anti-aggressive function, which was reversed by the *D160N* mutation, and appears to be different in different cell lines. In addition to increased invasion, there was also an increase in anchorage independent growth in mouse 4T1 cells. With no crystal structure currently available for SIVA, and based on a predictive model (http://www.sbg.bio.ic.ac.uk/), we can only hypothesize that the D160N mutation, located near/within the Zing-finger domain, may have disrupted a D160-K164 salt bridge, affecting the SIVA protein stability or specificity of the protein-protein interactions. However, exactly how the *SIVA-D160N* mutation affects the SIVA protein and its function, and how this perturbation by D160N mutation affects cancer metastasis is not yet known, and this is a limitation of the study. Protein molecules that can interact with this C-terminal segment of SIVA are worthy of further investigation with reduced complexity.

As SIVA is known to interact with multiple signaling molecules, its copy number reduction may have pleiotropic effects in cancer biology by affecting myriad interactions simultaneously. In this regard, *SIVA-D160N* mutation represented a rare opportunity when a circumscribed spectrum of pathways is expected to be affected by a single amino acid substitution, affording a unique opportunity to study the signaling pathway(s) governed by *D160N* in SIVA that mediated increased breast cancer aggressiveness *in vivo* and *in vitro*. Of note, over expression of wild type *SIVA* decreased rather than increased the apoptosis of 4T1 cells when introduced via orthotopic mammary fad pad injection in a syngeneic mouse model. This anti-apoptosis function was lost with the *D160N* mutation, suggesting an additional functional involvement of this segment in the suppression of apoptosis. This observation also suggested that the tumor aggressiveness promoting functions of *SIVA-D160N* in the 4T1 models was not associated with apoptosis.

Alteration in *SIVA* was rarely seen in primary breast cancer (less than 1% in TCGA). Deep deletion of *SIVA* was reported in 12% (36/301) of metastatic breast cancer, both receptor positive and receptor negative [50]. AKT1 was also deleted as AKT1 and *SIVA* genes were in close proximity on the chromosome 14. The serine/threonine kinase AKT is frequently hyperactivated in breast cancer through multiple mechanisms, including PI3K activation, *PTEN* loss, and ErbB2/Her2/neu activation/amplification [51]. Inducible ablation of *Akt1* in mammary epithelium after mammary tumors were formed inhibited tumor growth but not metastasis [36]. *AKT1* therefore did not appear to be the target for deletion while *SIVA* was. Taken together, these results indicate that the loss of *SIVA* function, via either deletion or *D160N* mutation, may promote breast cancer metastasis.

## Conclusions

Some of the bilateral breast cancers are clonally related, therefore representing breast-to-breast metastasis. Whole exome sequencing and comparative analysis of clonally related breast cancers from the same individual revealed that a very small number of somatic mutations was acquired in this breast-to-breast metastasis. One such mutation is D160N in *SIVA*. Our finding indicated that D160N functioned in a dominant negative function and promoted cancer spread in both *in vitro* and *in vivo* system. *SIVA* gene was deleted in 12% of metastatic breast cancer but *SIVA* aberration was rarely observed in primary breast cancer, suggesting a role of *SIVA* in cancer metastasis. Further characterization and understanding of SIVA function, and that of its interacting proteins, may elucidate mechanisms of breast cancer metastasis, providing clinically useful biomarkers and therapeutic targets.

## Supporting information

S1 File Uncropped images of Western blots. (PDF)

S2 File Supporting figures and tables. (PDF)

S3 File Supporting RNAseq methodology and results. (PDF)
