# Supplementary material for: Mutation of SIVA, a candidate metastasis gene identified from clonally related bilateral breast cancers, promotes breast cancer cell spread in vitro and in vivo
Source: PLoS One. 2024 May 9;19(5):e0302856. doi: 10.1371/journal.pone.0302856 (PMC11081324; doi:10.1371/journal.pone.0302856)
Supplement: S1 File — (PDF) [file pone.0302856.s001.pdf]

Fig 2B

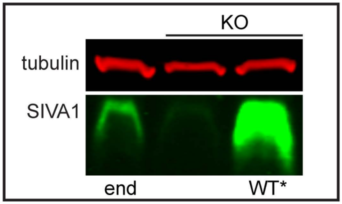

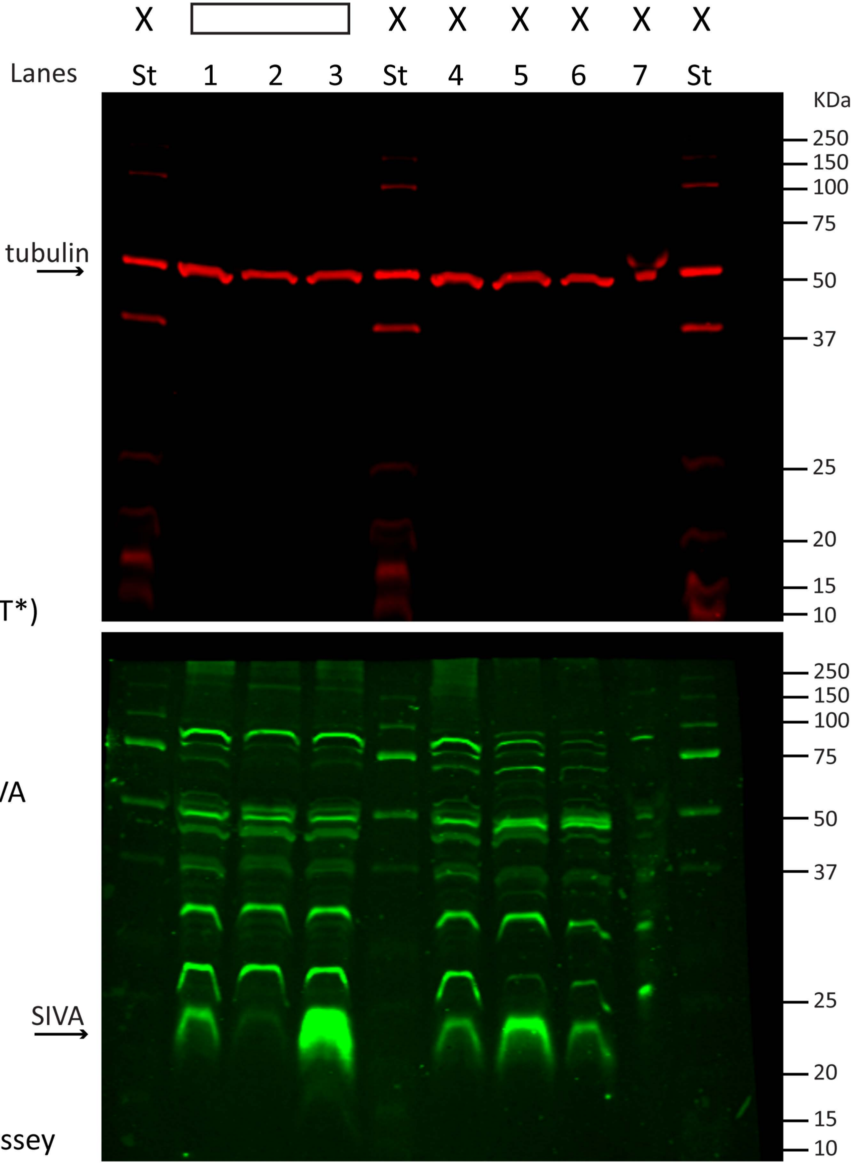

St: standard

1: 231endogenous SIVA (end)

2: 231 SIVA knockout (KO)

3: 231 SIVA-KO + SIVA-WT (WT*)

4: 231 endogenous SIVA

5: MCF7 endogenous SIVA

6: SKBR3 endogenous SIVA

7: mouse 4T1 endogenous SIVA

One blot

Tubulin (red, top)

SIVA (green, bottom)

Images taken with LI-COR Odyssey

Fig 2E

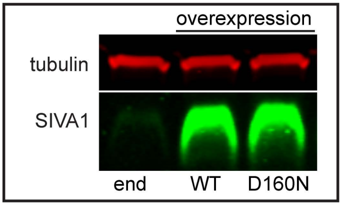

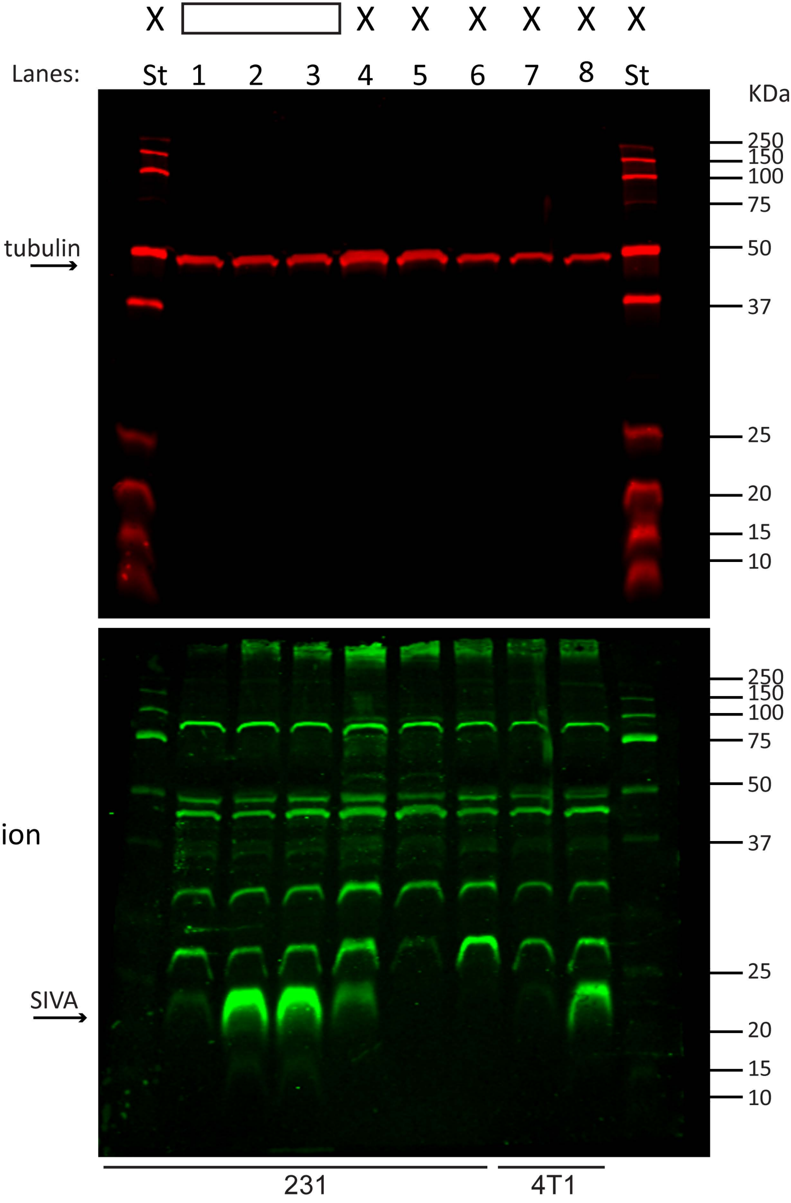

St: standard

1: 231 empty vector (SIVA endogenous)

2: 231 SIVA-WT overexpression

3: 231 SIVA-D160N overexpression

4: 231 SIVA siRNA (random)

5: 231 SIVA siRNA (09)

6: 231 SIVA siRNA (12)

7: mouse 4T1 SIVA-WT overexpression

8: mouse 4T1 SIVA-D160NT overexpression

One blot

Tubulin (red, top)

SIVA (green, bottom)

Images taken with LI-COR Odyssey

Fig 3A

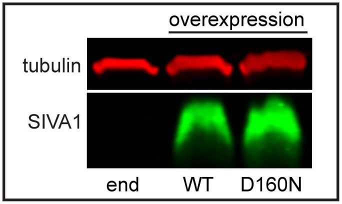

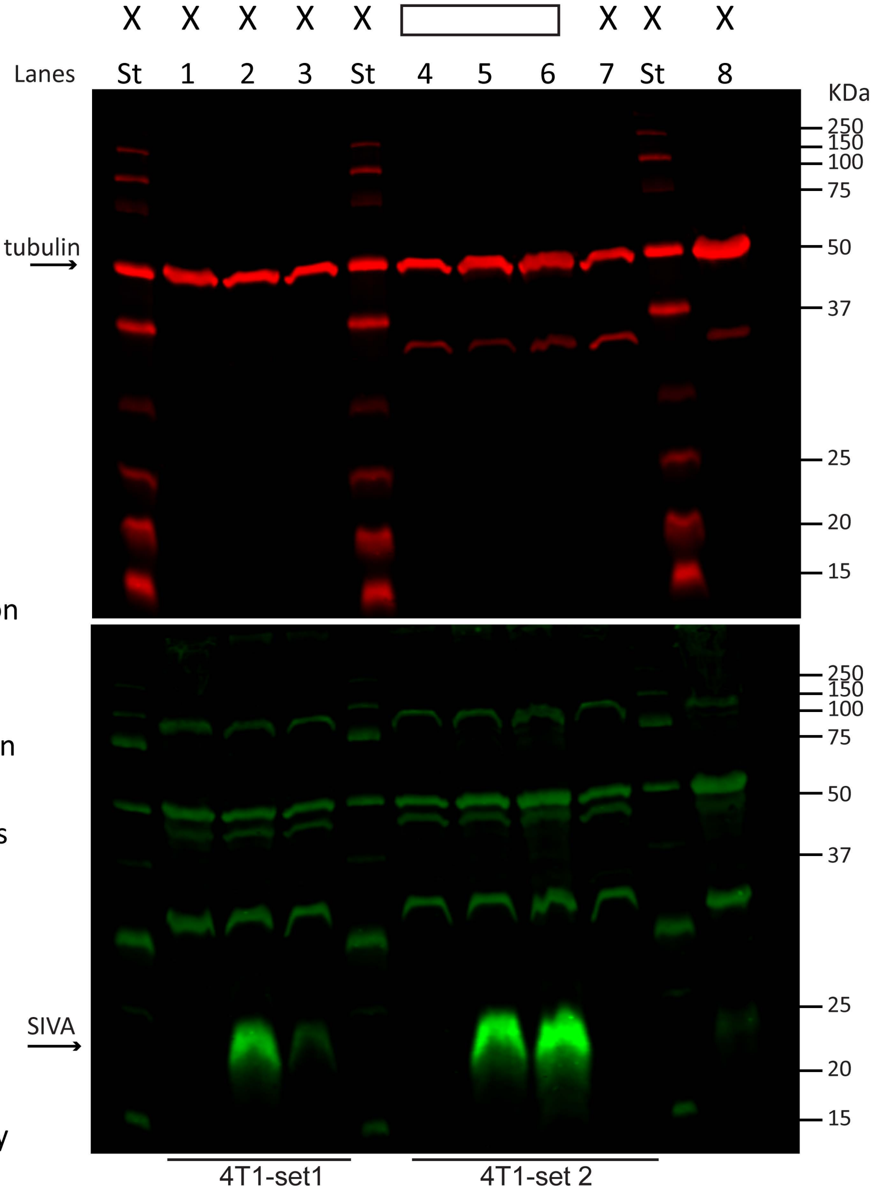

St: standard

1: 4T1 empty vector (SIVA end)

2: 4T1 SIVA-WT overexpression

3: 4T1 SIVA-D160N overexpression

4: 4T1 empty vector (SIVA end)

5: 4T1 SIVA-WT overexpression

6: 4T1 SIVA-D160N overexpression

7: 4T1 empty vector (SIVA end)

8: human LM24 SIVA endogenous

One blot

Tubulin (red, top)

SIVA (green, bottom)

Images taken with LI-COR Odyssey

S Fig 2A

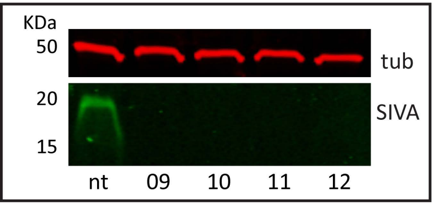

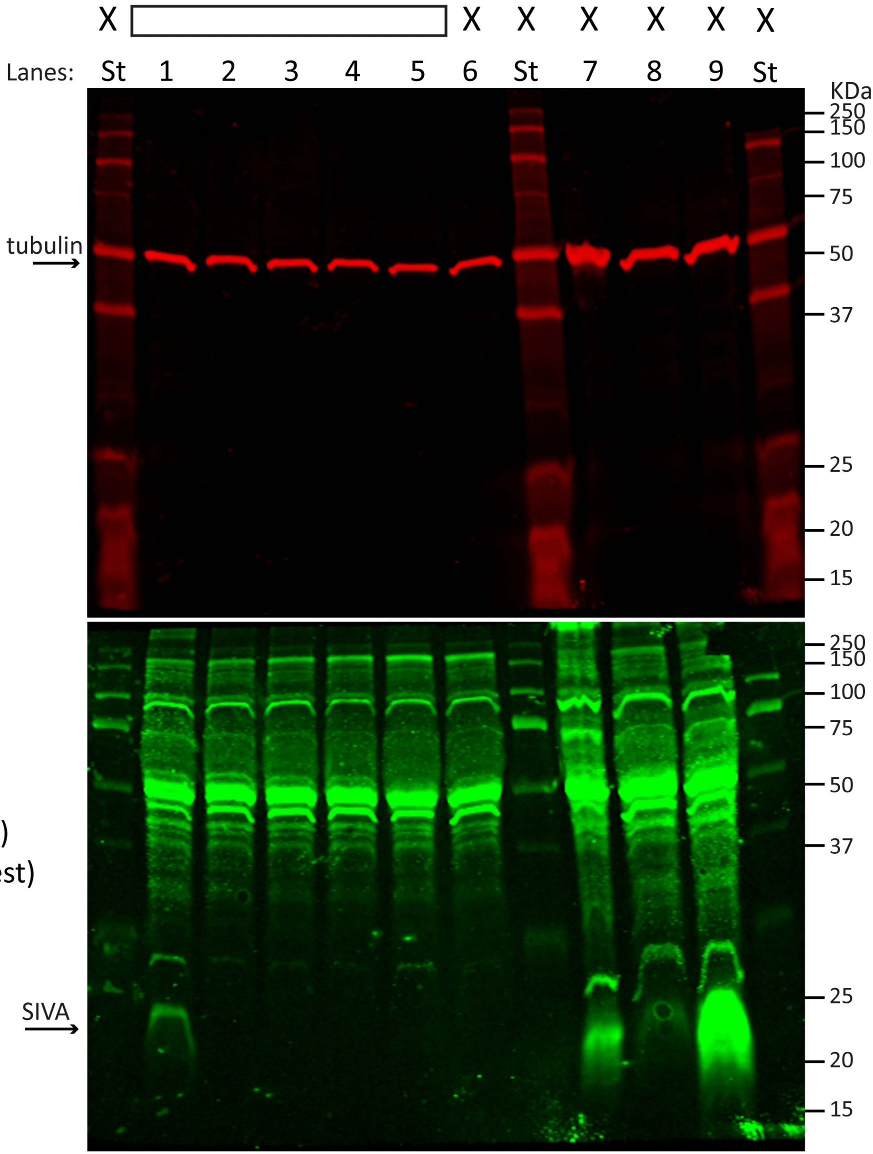

St: standard

1: 231 siRNA non-targeting (nt)

2: 231 siRNA (09)

3: 231 siRNA (10)

4: 231 siRNA (11)

5: 231 siRNA (12)

6: 231 siRNA (09+12)

7: 231 SIVA endogenous

8: 231 $SIVA^{WT}$ overexpression (test)

9: 231 $SIVA^{D160N}$ overexpression (test)

One blot

Tubulin (red, top)

SIVA (green, bottom)

Images taken with LI-COR Odyssey

S Fig 5A

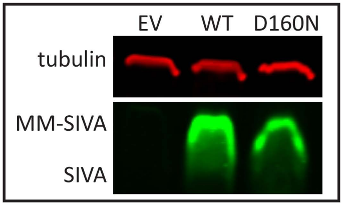

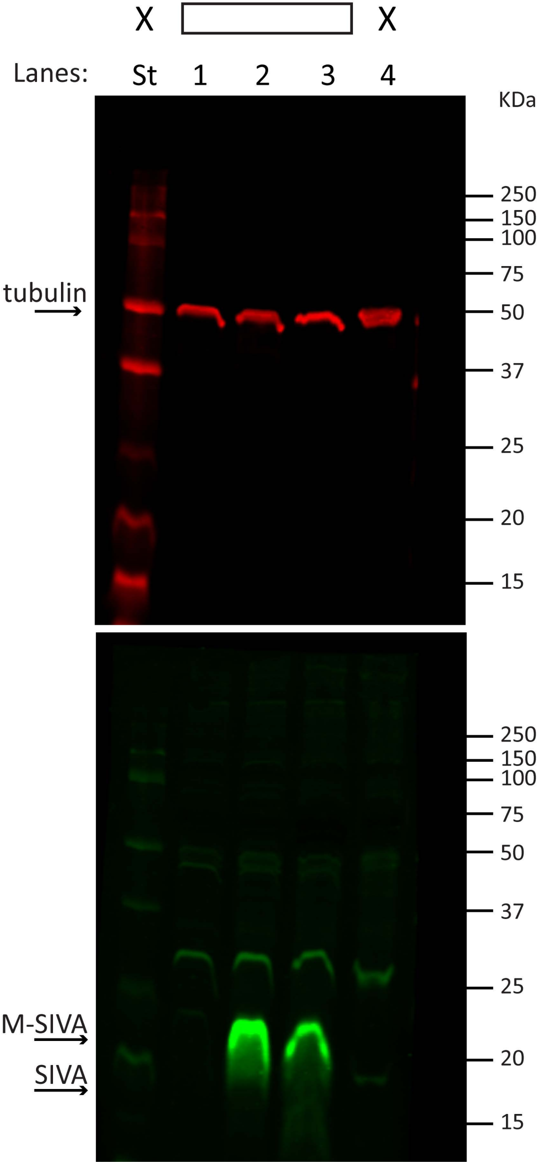

St: standard

1: HCC1954 empty vector (EV)

2: HCC1954 + SIVA-WT overexpression

3: HCC1954 + SIVA-D160N overexpression

4: HCC1954 + SIVA knockout

One blot

Tubulin (red, top)

SIVA (green, bottom)

Images taken with LI-COR Odyssey

S Fig 5B

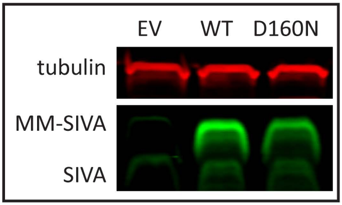

St: standard

1: SKOV3 empty vector (EV)

2: SKOV3 + SIVA-WT overexpression

3: SKOV3 + SIVA-D160N overexpression

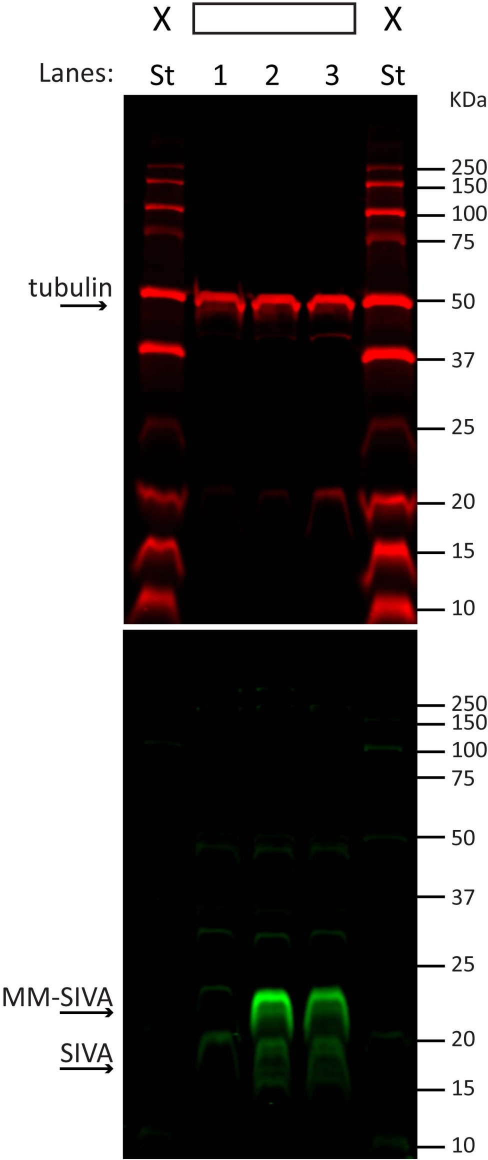

One blot

Tubulin (red, top)

SIVA (green, bottom)

Images taken with LI-COR Odyssey

S Fig 5C

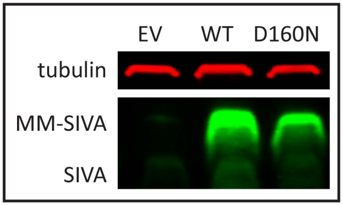

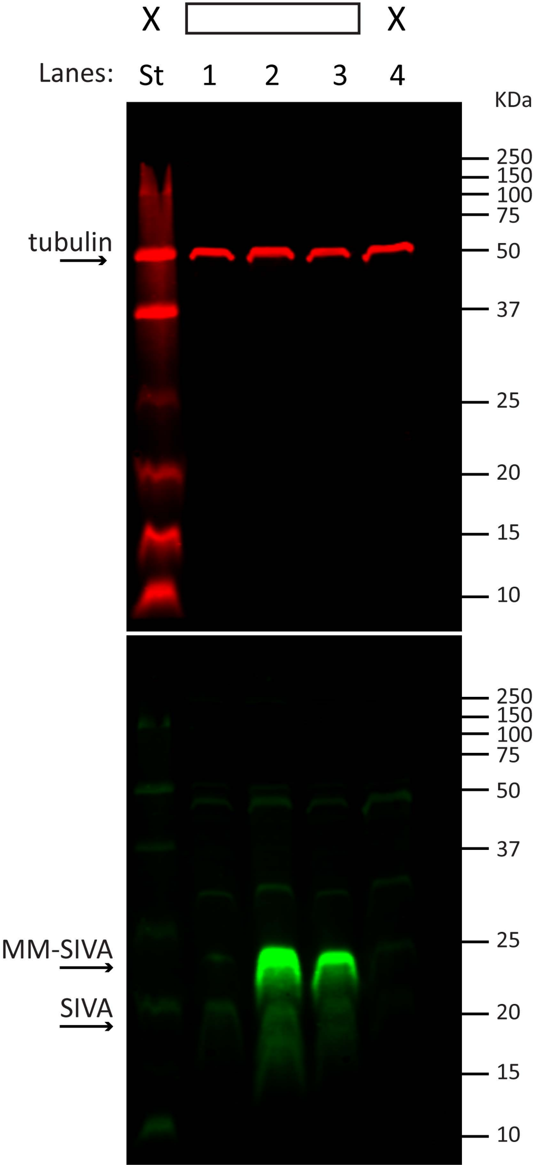

St: standard

1: OVCAR8 empty vector (EV)

2: OVCAR8 + SIVA-WT overexpression

3: OVCAR8 + SIVA-D160N overexpression

4: OVCAR8 + SIVA knockout

One blot

Tubulin (red, top)

SIVA (green, bottom)

Images taken with LI-COR Odyssey
