# Supplementary material for: Mutation of SIVA, a candidate metastasis gene identified from clonally related bilateral breast cancers, promotes breast cancer cell spread in vitro and in vivo
Source: PLoS One. 2024 May 9;19(5):e0302856. doi: 10.1371/journal.pone.0302856 (PMC11081324; doi:10.1371/journal.pone.0302856)

ADDITIONAL FILES

S1 Fig. Target coverage for tumor sets by whole exome sequencing (WES). 16 bilateral tumor samples (16 pairs = 32 total). Lowest coverage was 79x.

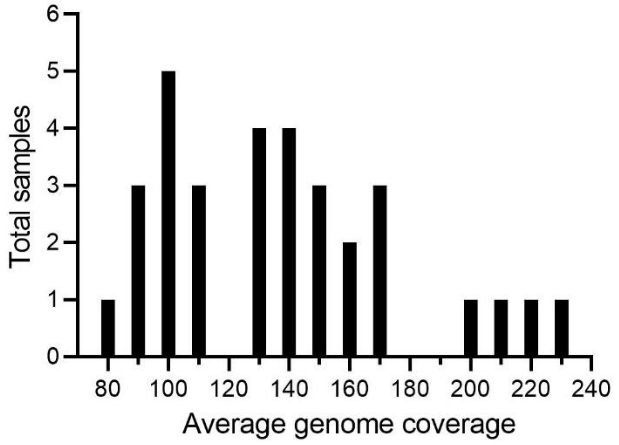

Table A. Counts of samples with a mutation in the indicated gene along with the fraction of our samples and the fraction of TCGA-BRCA samples with that gene mutated.

Gene	Total Mutations in our 32 samples	Fraction of our sample with gene mutated	Fraction of TCGA-BRCA samples with mutated gene
PIK3CA	11	0.34	0.34
MLL3	1	0.03	0.09
CDH1	13	0.41	0.14
MAP3K1	5	0.16	0.09
TP53	5	0.16	0.34
FOXA1	3	0.09	0.03
ARID1A	1	0.03	0.04
SF3B1	1	0.03	0.02
GATA3	1	0.03	0.1
PTEN	1	0.03	0.05
RPGR	4	0.13	0.02
CASP8	6	0.19	0.02
KDM6A	1	0.03	0.02

Table B. *PIK3CA* calls from 32 breast cancer tumors, called using R vs L tumor

Tumor	Gene	aa change	chr	Start	Ref	Alt
24	*PIK3CA*	p.H1047R	3	178952085	A	G
14RT	*PIK3CA*	p.E542K	3	178936082	G	A
1563	*PIK3CA*	p.H1047R	3	178952085	A	G
159	*PIK3CA*	p.H1047R	3	178952085	A	G
218	*PIK3CA*	p.H1047R	3	178952085	A	G
21L	*PIK3CA*	p.M1043I	3	178952074	G	A
608	*PIK3CA*	p.H1047R	3	178952085	A	G

S2 Figure. Predictive alphafold model of SIVA protein. SIVA D160 is located in the Zing-finger domain (left), possibly forming a D160-K164 salt bridge (right) that could be disrupted by the D160N mutation (www.sbg.bio.ic.ac.uk/).

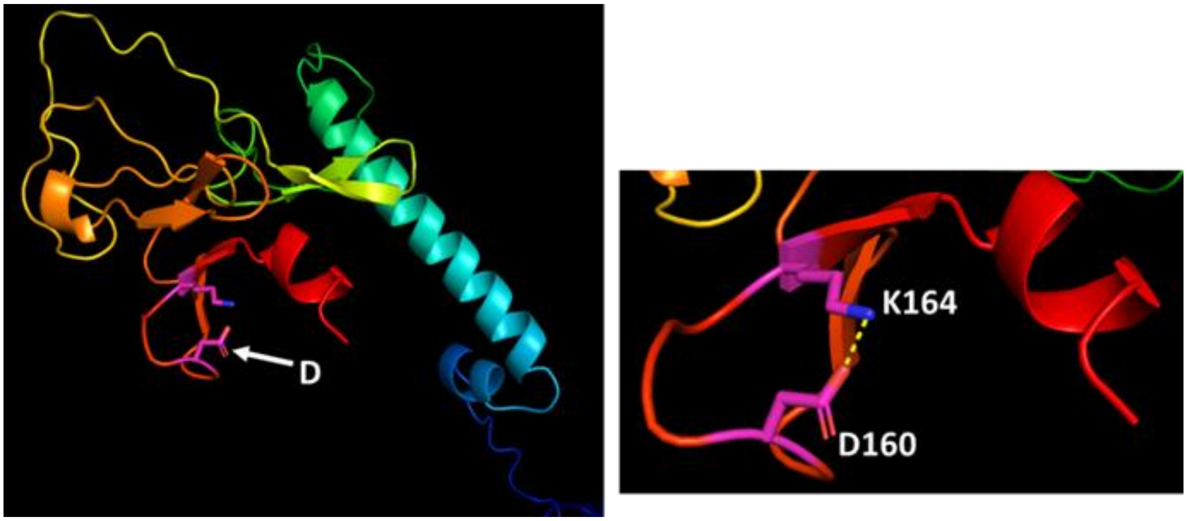

Supplemental Figure S3. Transient downregulation of *SIVA1* significantly enhanced cell migration and invasion. (**A**) Transient downregulation of SIVA1 protein expression by four different siRNA sequences (09, 10, 11, and 12) was demonstrated by Western analysis. 09 and 12 were chosen for further analysis. (**B**) siRNA sequences; nt=non-targeting control siRNA. (**C**) Cell migration and (**D**) cell invasion were examined in MB-MDA-231L cells transiently expressing non-targeting, 09, 12 and 09+12 siRNA constructs, using the Boyden Chamber (43500 cells seeded/insert, 6h incubation) and Matrigel matrix (37500 cells seeded/insert, 24h incubation) invasion assays respectively. Each assay was performed four times (n=4, total cells in 4 images/assay) with identical results. Analyses were expressed as cell counts per 4 fields of view (4 fov) normalized against EV control. Each bar represents mean ± SEM. One-way ANOVA (**C**: $p<0.0001$; **D**: $p=0.0002$) was followed by a Dunnett's post-hoc test (*$p<0.05$, **$p<0.01$, ***$p<0.001$, ****$p<0.0001$). Below each graph are representative images of the migration and invasion assay inserts following staining with Crystal violet.

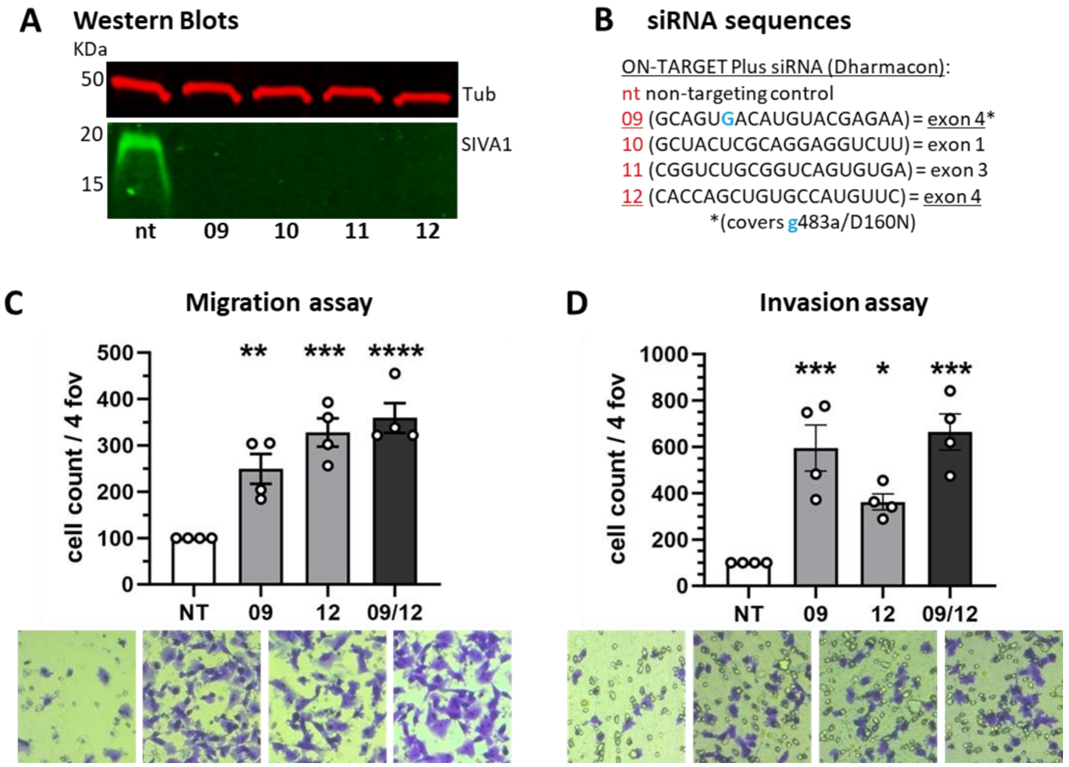

Supplemental Figure S4. Cultured *SIVA1-160N* expressing 4T1 cells formed significantly more aggregates. Representative images showing presence of cell aggregates in *SIVA1-D160N* expressing 4T1 cells that persisted upon reseeding after filtering trypsinized cells to remove aggregates (H&E). Scale bar 500 µm.

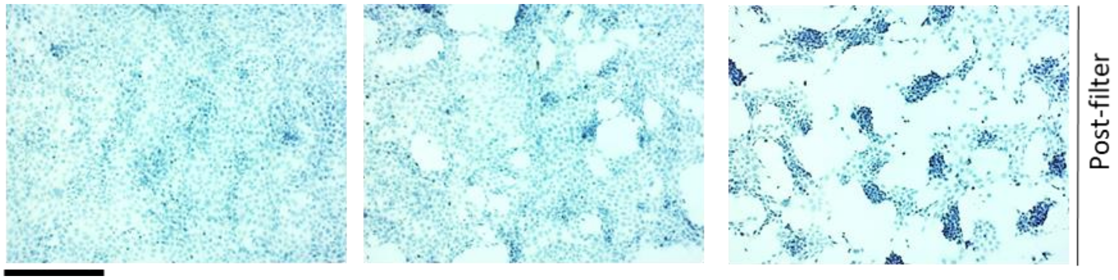

Supplemental Figure S5. Representative images from Ki67, TUNEL and H&E stainings of tumors from mice injected with mouse 4T1 cells expressing empty vector (EV, control), *SIVA1-WT* (WT) or *SIVA1-D160N* (D160N). (A) Representative images of tumors stained with Ki67 to monitor active cell proliferation, scale bar 150 µm. **(B)** Representative images of tumors stained with TUNEL assay to monitor apoptosis, scale bar 150 µm. **(C)** Representative images showing METs in liver sections. LEFT: 10 and 16 cells/MET, scale bar 35 µm, RIGHT: liver section showing three METs, 150 µm).

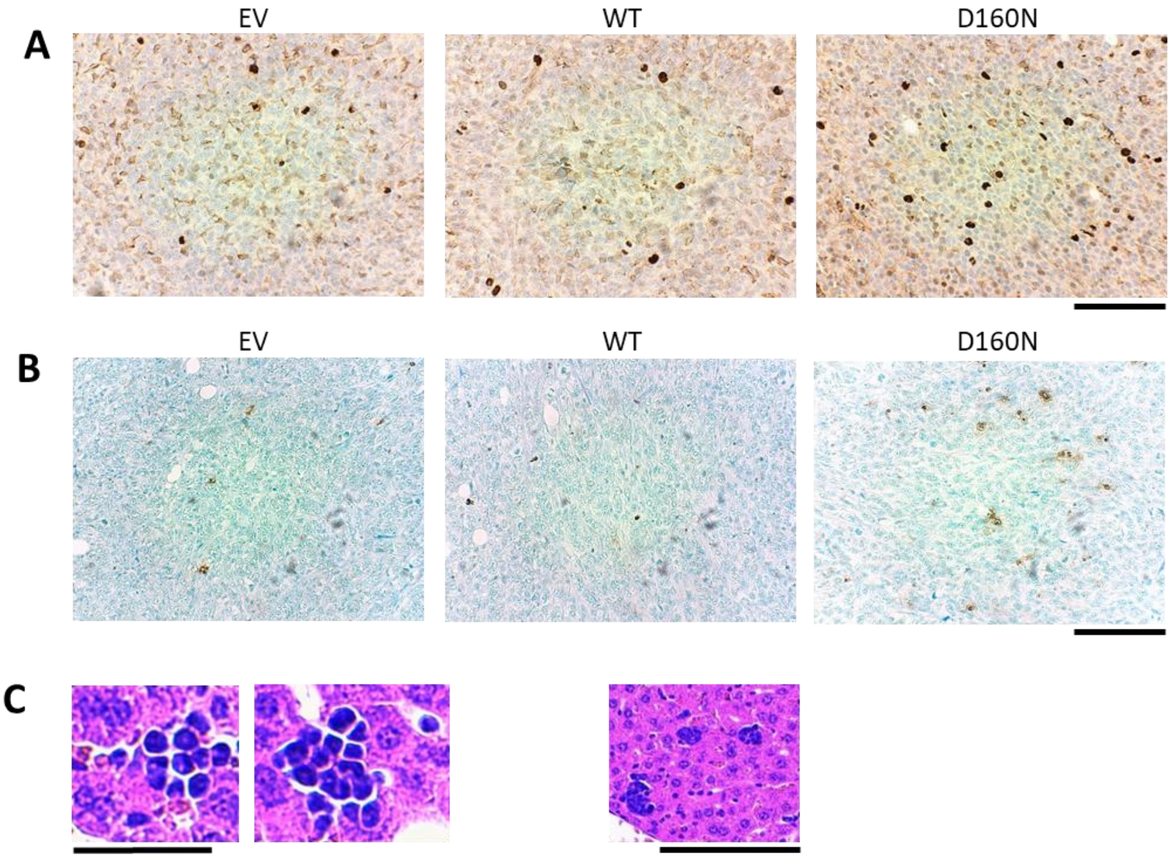

Supplemental Figure S6. Over-expression of SIVA1-WT and SIVA1-D160N proteins in HCC1954, SKOV3 and OVCAR8 cell lines, and their impact on cell migration and invasion. (A) Western analysis showed overexpression of WT and D160N SIVA1 proteins compared to empty vector (EV) control, probed with anti-tubulin and anti-SIVA1 antibodies. **(B)** Migration assays of breast cancer (HCC1954, $p<0.0001$) and ovarian (SKOV3, $p=0.0084$; OVCAR8, $p=0.0002$) cell lines expressing EV, *SIVA1-WT* or *SIVA1-D160N*. Analyses were expressed as total cell counts in 4 fields of view (4 fov) normalized against EV control. In all cases, migration of SIVA1-WT expressing cells were significantly lower compared to EV control cells, with the breast cancer cell line (HCC1954) showing the biggest reduction. D160N expressing cells were all significantly higher compared to WT, as well as HCC1954 and OVCAR8 EV controls. **(C)** Invasion assays of breast cancer (HCC1954, $p<0.0001$) and ovarian (SKOV3, $p=0.0073$; OVCAR8, $p=0.0005$) cell lines expressing EV, *SIVA1-WT* or *SIVA1-D160N*. Analyses were expressed as total cell counts in 4 fields of view (4 fov) normalized against EV control. In all cases, invasion of *SIVA1-D160N* expressing cells were significantly higher compared to WT and EV controls, SIVA1-WT was significantly lower from EV control in HCC1954 breast cancer cell lines. Each bar represents the mean ± SEM (n=3, total cells in 4 images/assay). Statistical tests: one-way ANOVA, followed by a Tukey's multiple comparisons test, *$p<0.05$, **$p<0.01$. ***$p<0.001$, ****$p<0.0001$.

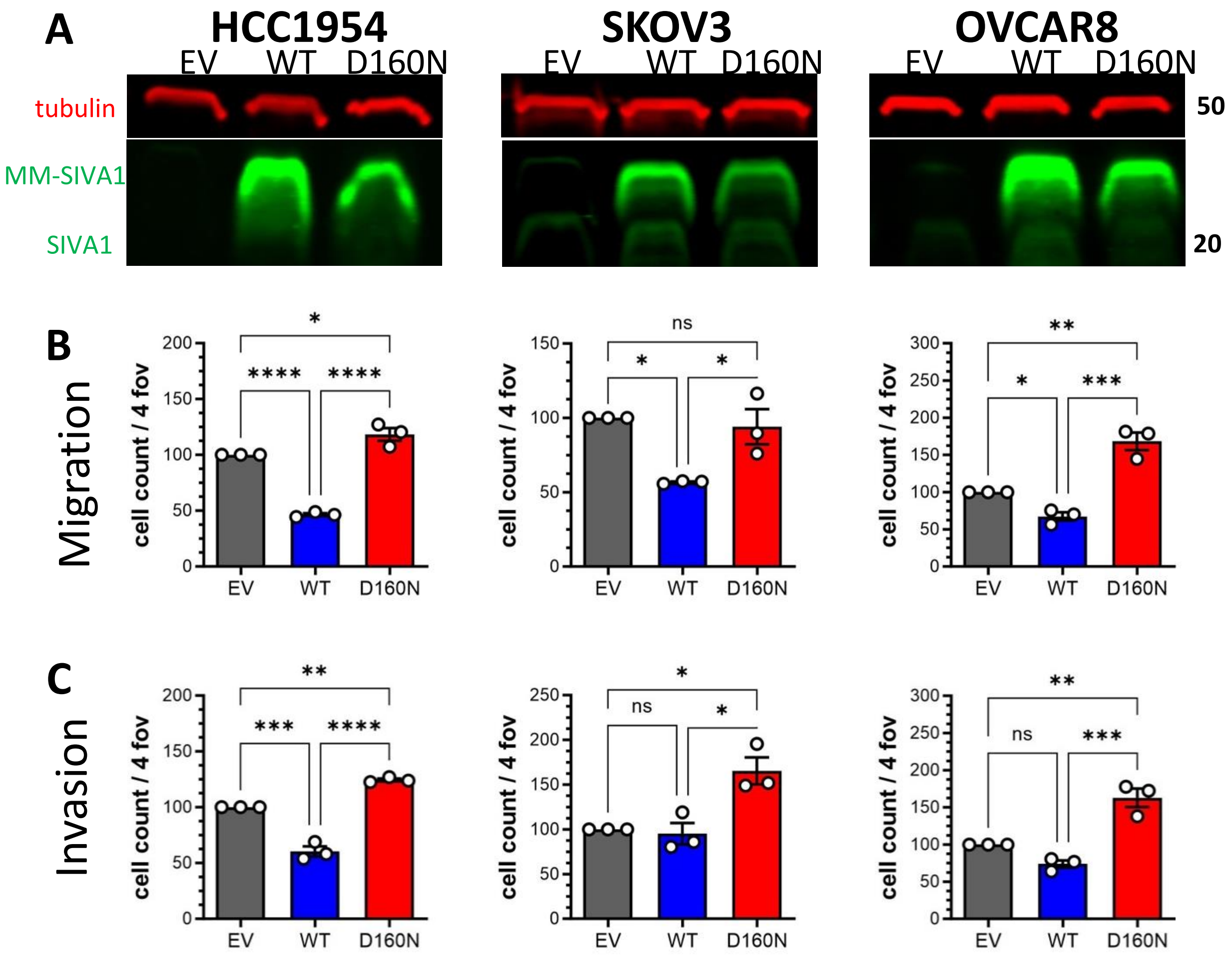

Supplement: S2 File — (PDF) [file pone.0302856.s002.pdf]
