# Supplementary material for: Mutation of SIVA, a candidate metastasis gene identified from clonally related bilateral breast cancers, promotes breast cancer cell spread in vitro and in vivo
Source: PLoS One. 2024 May 9;19(5):e0302856. doi: 10.1371/journal.pone.0302856 (PMC11081324; doi:10.1371/journal.pone.0302856)
Supplement: S3 File — (PDF) [file pone.0302856.s003.pdf]

RNAseq analysis of different cancer cell lines

Material and Methods.

Total RNA was purified using the Trizol reagent, and integrity was determined by running samples on the Bioanalyzer (Agilent, Santa Clara, CA, USA). Libraries were prepared using the TruSeq Stranded mRNA kit (Illumina, San Diego, CA, USA). Briefly, RNA was converted to cDNA using random hexamers. Synthesis of the second strand was done with the addition of dUTP, which enforced the stranded orientation of the libraries by blocking amplification off the second strand during the first round of PCR. Amplified libraries were profiled on the 4200 TapeStation (Agilent, Santa Clara, CA, USA). Libraries were quantified for sequencing using an NGS Library Quantification Kit (Roche/Kapa Biosystems, Indianapolis, IN, USA) on a StepOnePlus Real Time PCR Workstation (Thermo/ABI, Waltham, MA, USA). Sequencing was done on a NovaSeq 6000 (Illumina, San Diego, CA, USA). Fastq files generated from base-call files using bcl2fastq (Illumina, San Diego, CA, USA). Paired-end, 100-cycle RNA sequence was performed on an Illumina Hi-Seq 2500 sequencer at a depth of approximately 200X. Satisfactory integrity of the resultant FASTQ files was be assured using FastQC [34], following which, reads were aligned to the GRCh38 human genome reference utilizing the STAR alignment algorithm [35]. Gene counts were extracted from the STAR results and input into the edgeR R package [36] where non-expressing genes and genes lacking an EntrezGene ID were filtered out, library sizes adjusted, TMM (trimmed mean of M values) normalization [37] was performed, and logged counts per million (logCPM) calculated. Significant differential expression was determined by a log2 fold change of 1.5 and a false discovery rate of less than 0.05 (FDR < 0.05), as assessed by fitting count data to a quasi-likelihood negative binomial generalized log-linear model. Gene set enrichment analysis and functional annotation using the Gene Set Enrichment Analysis (GSEA, RRID:SCR_003199) algorithm ([38] and the GOnet web annotation tool [39, 40] were applied to identify any potential therapeutic targets present within the gene set and/or genes associated with cell migration and invasion pathways.

The GSEA v4.1.0 application was run on our gene list against the entire Molecular Signatures Database of gene sets using the msigdb.v5.1.symbols.gmt and GENE_SYMBOL.chip files downloaded from [41]. All other parameters were left at their default settings. Significantly enriched gene sets were retained based on an FDR value of less than 0.25, as recommended in the GSEA documentation. GO term enriched was performed on our gene list with the GOnet web application (ontology version: 2019-07-01; human annotation version: 2019-07-01) with parameters set at default levels. Resulting enriched GO term gene sets were filtered for FDR less than 0.05 and gene set size greater than 5 genes and less than 100 genes.

Prioritization of GSEA and GO results begun with filtering the respective results such that redundancy between gene set hits was reduced as much as possible. To this end, enriched sets/terms were hierarchically clustered using the Ward D2 method (Ward's Hierarchical Agglomerative Clustering Method) to highlight those sets/terms with an abundance of overlapping gene names. The most representative term, or terms, of each cluster was selected using a heuristic approach centered on a combination of FDR significance, abundance of other functionally related terms within the cluster, and where relevant, position of the term within the GO hierarchical tree. In respect to this latter criterium, preference was given toward those terms residing lowest (i.e., closest to the "leaves") in the tree.

Results.

In light of our work showing the SIVA-D160N mutation facilitates cancer cell migration and invasion, we have generated RNA sequence from a cohort of twelve cell lines in order to identify aberrant signaling pathways. With this sequence, we performed differential gene expression analysis between OVCAR8, SKOV3, HCC1954, MBA-MB-231 parental cell lines (EV) and their SIVA-WT and SIVA-D160N transformed counterparts. Contrasting gene expression in control cell lines (expressing empty expression vectors (EV)) with the corresponding SIVA-WT overexpressing cells did not yield reportable results. Similarly, neither did comparing the gene expression between the SIVA-WT and SIVA-D160N overexpressing cell lines. Approximately 91% and 87% of differentially expressed genes identified in comparisons of SIVA-WT vs SIVA-D160N and SIVA-WT vs parental controls (EV), respectively, overlapped with those genes

differentially expressed in SIVA-D160N vs EV. Hence, we decided to restrict our reported findings to the 466 genes identified from the contrast of SIVA-D160N and the parental cell lines, as parallel gene expression analyses of SIVA-WT vs SIVA-D160N and SIVA-WT vs EV produced no additional findings of significance.

Differential expression of 466 genes is noted between the SIVA-D160N and parental cell lines. The pathways most closely represented by these genes are shown in supplemental Table S3 Table. We used the NDEx Integrated Query web tool [52] to perform integrated analyses with the results presented in the SIVA knockout lung cancer models publication [53]. Several noteworthy pathways associated with SIVA depletion/SIVA dominant negative interference were reported, including:

1. Increased activity of the HIPPO-YAP signaling pathway where YAP activates the PI3K-mTOR pathway through inhibition of PTEN, resulting in increased cell proliferation and a decrease in apoptosis.
2. Increased autophagy felt to be due to a decreased phosphorylation and inactivation of ULK1.
3. Negative regulation of mitogenic signaling pathways, such as ERK1/2, WNT, and AKT signaling.

Our gene list had some overlap in pathways associated with invasion and metastasis, PI3K-AKT-mTOR signaling, PI3K-AKT signaling, senescence and autophagy in cancer, and HIPPO signaling regulation. Most overlapping genes are associated with more than one of these pathways. Since our gene set expression analysis did not reach statistical significance, our findings are considered exploratory in nature.

Table. Pathway enrichment using the 466 genes differentially expressed between parental cell lines (EV) and their *SIVA1-D160N* mutant counterparts suggest a modest overlap (p-value = 0.377) with curated pathways (e.g., WikiPathways, NCI-PID, etc) of interest.

Curated pathways	Overlapped genes / total	genes
Activating invasion and metastasis (CPTAC)	22 / 541	ATF4, CAV1, CDH16, CDKN1A, COL3A1, DDIT4, EPAS1, FGF21, FOS, GDF15, HSP90AA1, IL74, KIT, KITLG, MAPK13, NGF, RELN, SPP1, SREBF1, THBS1, THBS3, ULK1
Focal adhesions PI3K-Akt-mTOR-signaling pathway (WP3932)	17 / 303	ATF4, CDKN1A, COL3A1, DDIT4, EPAS1, FGF21, HSP90AA1, IL74, KIT, KITLG, NGF, RELN, SSP1, SREBF1, THBS1, THBS3, ULK1
PI3K-AKT signaling (WP4172)	17 / 339	ATF4, CCNE2, CDK6, CDKN1A, DDIT4, FGF21, FGF5, HSP90AA1, IL7R, KIT, KITLG, NGF, PCK2, RELN, SPP1, THBS1, THBS3
Senescence and autophagy in cancer (WP615)	10 / 114	CDKN1, COL3A1, GABARAPL1, IL24, INHBA, MMP14, SERPINB2, SERPINE1, THBS1, ULK1
Hippo signaling regulation (WP4540)	3 / 98	CDH16, KIT, PLCB4
